# Small molecule Y-320 stimulates ribosome biogenesis, protein synthesis, and aminoglycoside-induced premature termination codon readthrough

**DOI:** 10.1371/journal.pbio.3001221

**Published:** 2021-05-03

**Authors:** Sara Hosseini-Farahabadi, Alireza Baradaran-Heravi, Carla Zimmerman, Kunho Choi, Stephane Flibotte, Michel Roberge

**Affiliations:** 1 Department of Biochemistry and Molecular Biology, University of British Columbia, Vancouver, British Columbia, Canada; 2 UBC/LSI Bioinformatics Facility, University of British Columbia, Vancouver, British Columbia, Canada; Stanford University, UNITED STATES

## Abstract

Premature termination codons (PTC) cause over 10% of genetic disease cases. Some aminoglycosides that bind to the ribosome decoding center can induce PTC readthrough and restore low levels of full-length functional proteins. However, concomitant inhibition of protein synthesis limits the extent of PTC readthrough that can be achieved by aminoglycosides like G418. Using a cell-based screen, we identified a small molecule, the phenylpyrazoleanilide Y-320, that potently enhances *TP53*, *DMD*, and C*OL17A1* PTC readthrough by G418. Unexpectedly, Y-320 increased cellular protein levels and protein synthesis, measured by SYPRO Ruby protein staining and puromycin labeling, as well as ribosome biogenesis measured using antibodies to rRNA and ribosomal protein S6. Y-320 did not increase the rate of translation elongation and it exerted its effects independently of mTOR signaling. At the single cell level, exposure to Y-320 and G418 increased ribosome content and protein synthesis which correlated strongly with PTC readthrough. As a single agent, Y-320 did not affect translation fidelity measured using a luciferase reporter gene but it enhanced misincorporation by G418. RNA-seq data showed that Y-320 up-regulated the expression of CXC chemokines CXCL10, CXCL8, CXCL2, CXCL11, CXCL3, CXCL1, and CXCL16. Several of these chemokines exert their cellular effects through the receptor CXCR2 and the CXCR2 antagonist SB225002 reduced cellular protein levels and PTC readthrough in cells exposed to Y-320 and G418. These data show that the self-limiting nature of PTC readthrough by G418 can be compensated by Y-320, a potent enhancer of PTC readthrough that increases ribosome biogenesis and protein synthesis. They also support a model whereby increased PTC readthrough is enabled by increased protein synthesis mediated by an autocrine chemokine signaling pathway. The findings also raise the possibility that inflammatory processes affect cellular propensity to readthrough agents and that immunomodulatory drugs like Y-320 might find application in PTC readthrough therapy.

## Abstract 2

A cell-based screen identifies a small molecule, Y-320, that potently enhances readthrough of premature termination codons by the aminoglycoside G418. Y-320 increases cellular protein levels and protein synthesis, and appears to act via an autocrine chemokine signaling pathway.

## Introduction

Nonsense mutations introduce a premature termination codon (PTC) into mRNA and underlie about 11% of genetic disorder cases [1]. Translation of PTC-bearing mRNAs leads to production of functionally defective truncated proteins. The relatively high incidence of nonsense mutations has stimulated extensive efforts to discover therapies known as nonsense suppression or PTC readthrough that aim to restore full-length protein production, in diseases such as Cystic Fibrosis and Duchenne Muscular Dystrophy (DMD) [2–5].

It has been known for several decades that certain aminoglycoside antibiotics can suppress nonsense mutations in yeast [6,7] and in mammalian cells [8,9]. They bind to the decoding center of cytosolic ribosomes and increase the frequency of pairing of near-cognate aminoacyl-tRNAs to the PTC, which leads to the formation of full-length protein [10,11]. However, aminoglycosides are far from ideal as readthrough drug candidates. They also bind to mitochondrial ribosomes and impairment of mitochondrial translation is considered a major cause of the toxicity of aminoglycosides to eukaryotic cells [12,13]. The design of PTC readthrough aminoglycosides with increased selectivity toward cytosolic ribosomes versus mitochondrial ribosomes may lead to better tolerated PTC readthrough drugs [14,15]. Nonetheless, relatively high concentrations of aminoglycosides are required for readthrough and it has been shown that aminoglycosides can bind to sites other than the decoding center at high concentrations, which may adversely affect protein synthesis [11].

This study further examines the relationship between induction of PTC readthrough and inhibition of protein synthesis. We show that at concentrations that induce PTC readthrough, the potent readthrough aminoglycoside G418 also inhibits protein synthesis, which limits the extent of readthrough that can be achieved. To study whether PTC readthrough can be increased without further inhibiting protein synthesis, we identify the phenylpyrazoleanilide Y-320 as a compound that does not affect readthrough when used as a single agent but that can enhance PTC readthrough when combined with G418. Interestingly, Y-320 enhanced readthrough without further inhibiting protein synthesis. Study of the mechanism of action of Y-320 shows it increases ribosome biogenesis and protein synthesis and that these processes are strongly correlated with enhanced readthrough at the single cell level. Y-320 appears to exert these effects by an autocrine mechanism involving increased expression of CXC chemokines.

## Results

### PTC readthrough by G418 is self limiting

**Fig 1:**
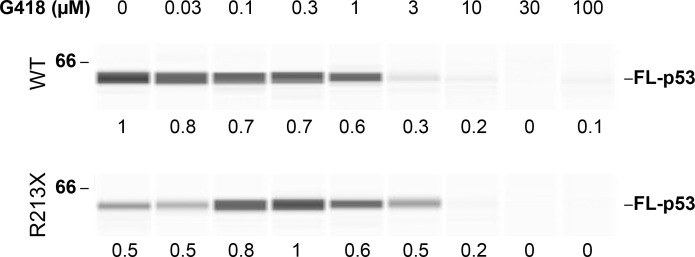
PTC readthrough in a cell-free translation assay. WT and R213X *TP53* mRNAs were translated in vitro in the presence of G418 at the indicated concentrations and equal amounts of protein were subjected to automated capillary electrophoresis western analysis. The data are displayed as “pseudo-blots” of bound p53 antibody chemiluminescence. FL-p53 chemiluminescence is expressed relative to the maximum value observed in either WT or R213X and shown under each lane. Uncropped images of automated capillary electrophoresis western analyses are provided in S1 Raw Images. FL-p53, full-length p53; PTC, premature termination codon; WT, wild-type.

Previous studies have shown that aminoglycosides such as G418 and gentamicin can enable PTC readthrough, leading to the synthesis of full-length proteins from mRNAs bearing nonsense mutations [7,8,10]. Paradoxically, their binding to ribosomes is also known to inhibit protein synthesis [10,11,15]. To examine the relationship between readthrough and protein synthesis during exposure to G418, we measured these processes in a cell-free translation assay using mRNA transcribed from wild-type (WT) *TP53* or *TP53* with a single base substitution leading to the R213X nonsense mutation. The 5′-capped and poly(A) tailed mRNAs were translated in vitro in a Hela cell lysate in the presence of different concentrations of G418. Synthesis of the full-length WT p53 protein, measured by automated capillary electrophoresis western analysis, was inhibited by G418 in a concentration-dependent manner (Fig 1 and S1 Fig). Some full-length p53 was produced from the R213X *TP53* mRNA in the absence of G418, reflecting spontaneous readthrough. G418 induced PTC readthrough, as seen by the increased production of full-length p53, which reached a peak at approximately 0.3 μM. However, higher G418 concentrations reduced readthrough, to the same extent as they inhibited the synthesis of WT p53 (Fig 1 and S1 Fig). This result indicates that inhibition of protein synthesis puts an intrinsic limitation on the extent of PTC readthrough that can be elicited by G418, making PTC readthrough by G418 a self-limiting process.

### Identification of Y-320 as a PTC readthrough enhancer

It has recently been shown that PTC readthrough by aminoglycosides can be enhanced by small molecules that do not themselves stimulate readthrough when used as single agents [16–19]. We hypothesized that compounds might exist that overcome the self-limitation and increase PTC readthrough by G418 without further inhibiting protein synthesis.

In order to discover such compounds, we assayed the Selleck bioactive library of 2658 compounds for enhancement of G418-induced PTC readthrough in HDQ-P1 cancer cells carrying homozygous *TP53* R213X nonsense mutations. Cells seeded in 96-well plates were exposed to the small molecules in combination with 50 μM G418. Although much higher than the concentration active in cell-free extracts, this G418 concentration is suboptimal in cell assays as the plasma membrane imposes a strong barrier to the intracellular diffusion of the highly polar aminoglycosides. After 72 h, *TP53* PTC readthrough was assessed using quantitative automated p53 immunofluorescence microscopy [17]. The phenylpyrazoleanilide Y-320 was the strongest PTC readthrough-enhancing compound (S1 Table). Y-320 is an immunomodulator shown to ameliorate type II collagen-induced arthritis in mice and monkeys by modulating IL-17 and other inflammatory markers, by an unknown mechanism [20].

**Fig 2:**
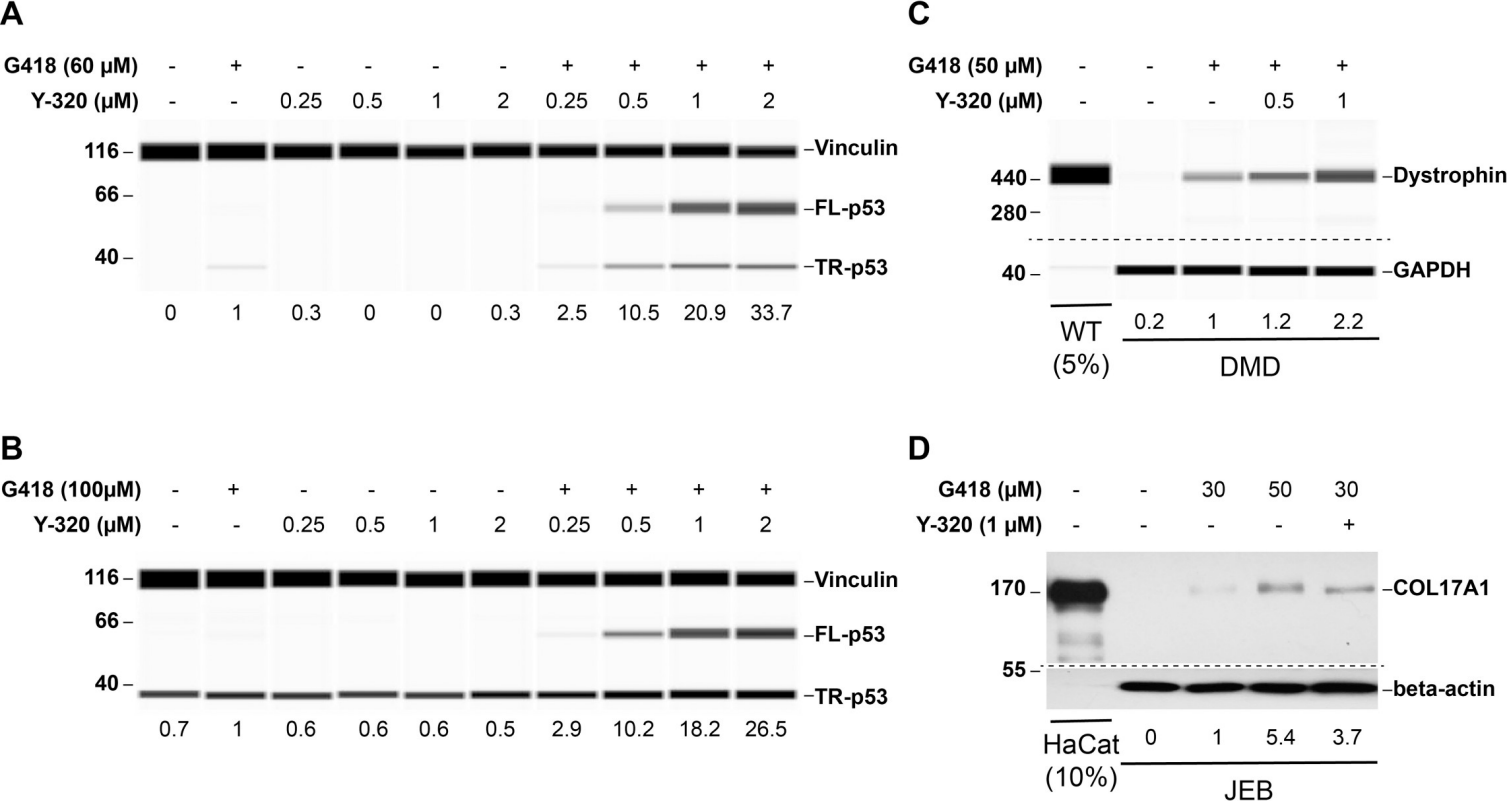
Y-320 enhances PTC readthrough by G418 in different cell lines. (A) p53 PTC readthrough in HDQ-P1 cells exposed to the indicated compounds for 48 h. Shown are pseudo blots of chemiluminescence of bound p53 and vinculin antibodies. The area under the FL-p53 peaks was first normalized to the vinculin loading control and then divided to that of G418 to provide lane-to-lane comparison. These numbers are displayed under the lanes. The MW ladder in kDa is shown at the left. (B) p53 PTC readthrough in H1299-R213X cells exposed to the indicated compounds for 24 h and imaged as in panel A. (C) Dystrophin PTC readthrough. WT and DMD patient-derived myoblasts were exposed to the indicated compounds in differentiating medium for 5 days. The proteins were separated using high-molecular weight capillaries. Shown are pseudo blots of bound dystrophin and GAPDH antibodies. The area under the dystrophin peaks was first normalized to the GAPDH loading control and then divided to that of G418 to provide lane-to-lane comparison. These numbers are displayed under the lanes. The amount of WT protein run in the capillary was 5% of that of DMD cells. (D) Collagen XVII PTC readthrough. Junctional Epidermolysis Bullosa patient-derived keratinocytes (JEB01) were exposed to the indicated concentrations of G418 and Y-320 for 72 h. COL17A1 protein levels were determined by traditional western blotting. The blots were scanned and COL17A1 band intensities were measured and normalized to the beta-actin loading control using ImageJ. The values are expressed relative to 30 μM G418 conditions to provide lane-to-lane comparison and are displayed under the lanes. The amount of HaCat protein run in the capillary was 10% of that of JEB cells. Dashed lines indicate different capillaries or blots. Uncropped images of automated capillary electrophoresis western analyses are provided in S1 Raw Images. DMD, Duchenne Muscular Dystrophy; FL-p53, full-length p53; GAPDH, glyceraldehyde-3-phosphate dehydrogenase; MW, molecular weight; PTC, premature termination codon; TR-p53, truncated p53; WT, wild-type.

To verify the screening result, HDQ-P1 cells were exposed to increasing concentrations of Y-320 alone or in combination with 60 μM G418 for 48 h. Cell lysates were subjected to automated capillary electrophoresis western analysis to quantitate full-length p53, the readthrough product. Untreated cells showed no full-length p53 (Fig 2A and S2 Fig), as did cells treated with Y-320 alone (S2 Fig). Exposure to G418 induced a low level of readthrough which was strongly enhanced by increasing concentrations of Y-320 (33-fold by 2 μM Y-320 + G418 compared to G418 alone; Fig 2A).

We also exposed HDQ-P1 cells to Y-320 alone or in combination with 300 μg/ml gentamicin sulfate for 72 h. Gentamicin did not elicit any readthrough but the combination induced full-length p53 (S2 Fig). We similarly measured the effect of Y-320 on *TP53* readthrough in H1299-R213X cancer cells, which do not express p53 because of homozygous deletion of the *TP53* gene and have been stably transfected with *TP53* R213X construct [16]. No full-length p53 was detected in untreated cells or cells treated with Y-320 alone. G418 induced p53 readthrough that was considerably enhanced by Y-320 (26-fold with 2 μM Y-320 + G418 compared to G418 alone; Fig 2B).

To determine whether Y-320 can also enhance the PTC readthrough of additional genes, we next tested it in DMD patient-derived myoblasts harboring a nonsense mutation in the *DMD* gene (E2035X). WT myoblasts, exposed to differentiation medium for 5 days to differentiate them into myotubes, expressed dystrophin, while no dystrophin expression was detectable in differentiated DMD myotubes (Fig 2C). Exposure of DMD cells to G418 during the differentiation period led to some dystrophin expression and cotreatment with Y-320 enhanced readthrough 2.2-fold over G418 alone (Fig 2C). The level of dystrophin in DMD cells exposed to 1 μM Y-320 + G418 was 1.4% of that of WT myoblasts. We also examined the effect of Y-320 on Junctional Epidermolysis Bullosa (JEB) keratinocytes carrying the homozygous R688X nonsense mutation in the *COL17A1* gene. HaCaT, a spontaneously transformed keratinocyte cell line from adult human skin was used as a control expressing WT *COL17A1*. HaCaT cells expressed a high level of COL17A1 protein which was not further increased by exposure to G418 alone or in combination with 1 μM Y-320 (S3 Fig). Untreated JEB cells showed no detectable signal (Fig 2D). At 30 μM, G418 induced detectable PTC readthrough which was enhanced 3.7-fold in combination with 1 μM Y-320, to about 0.06% of levels observed in HaCat cells (Fig 2D). These results show that Y-320 can enhance PTC readthrough by G418 in patient-derived cell types with nonsense mutations in different genes.

One possible mechanism for readthrough enhancement is stabilization of mRNAs bearing nonsense mutations via inhibition of nonsense mediated decay (NMD) [21,22]. However, exposure of HDQ-P1 cells to Y-320 alone did not significantly increase R213X *TP53* mRNA levels, indicating it is not an NMD inhibitor (S4 Fig). In combination with G418, Y-320 increased *TP53* mRNA levels up to 7-fold at 2 μM compared to G418 alone (S4 Fig), likely a consequence of increased PTC readthrough, which shields the mRNA from recognition by NMD [16,17]. Additionally, in the cell-free translation assay, G418 induced R213X *TP53* PTC readthrough but Y-320 did not enhance readthrough (S5 Fig), indicating that unlike G418, Y-320 likely does not directly target translating ribosomes.

### Effect of Y-320 on protein synthesis

**Fig 3:**
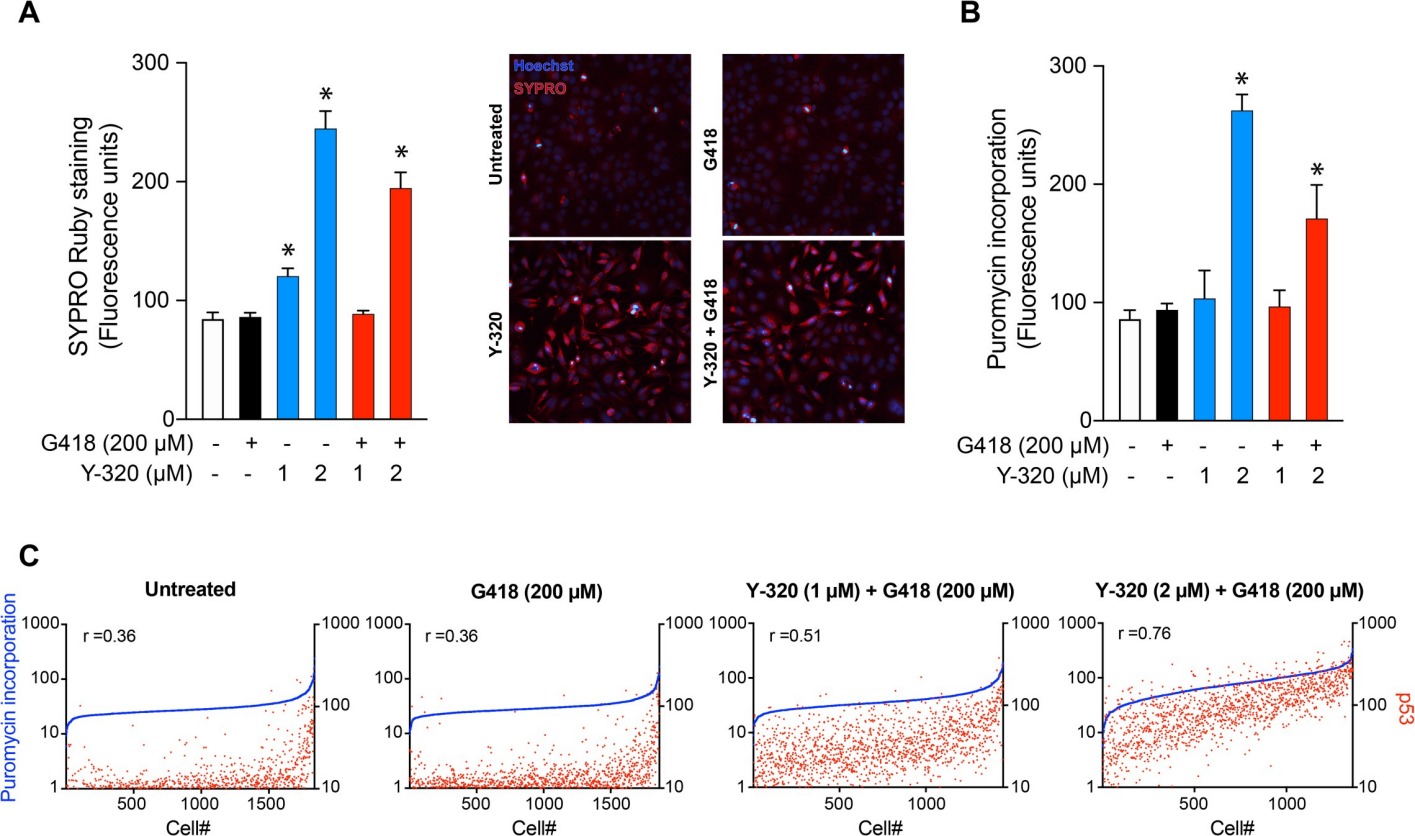
Stimulation of protein synthesis by Y-320 and correlation with p53 readthrough at the single cell level. (A) SYPRO Ruby protein staining. HDQ-P1 cells treated with the indicated compounds for 48 h were stained using SYPRO Ruby. Data were collected by automated fluorescence microscopy. Representative images are shown with SYPRO Ruby stain in red and nuclei in blue. (B) Puromycin detection of protein synthesis. Treated HDQ-P1 cells were exposed to puromycin and labeled with anti-puromycin AF488 antibody. Data were collected by automated fluorescence microscopy. In panels A and B, asterisks show statistically significant differences between treated and untreated cells (*p*-value < 0.05; mean ± SD; *n =* 4). (C) Quantitation of puromycin and p53 in single cells. HDQ-P1 cells treated with the indicated compounds were exposed to 2 μg/ml harringtonine and 10 μg/ml puromycin for 10 min. Cells were labeled with anti-puromycin AF488 and anti-p53 AF594 antibodies. The graphs are representative of each condition (*n* = 4). Red dots (right *y* axis) represent nuclear p53 expression and blue dots (left *y* axis) show cellular puromycin labeling. The Spearman rank correlation coefficient (r) for each condition is shown in each graph. The numerical data underlying the plots can be found in S1 Data.

The increased readthrough activity of aminoglycosides has been shown to correlate with their inhibitory effects on protein synthesis [14]. Having discovered that Y-320 can enhance PTC readthrough, we asked whether it would adversely affect cellular protein levels or protein synthesis. We examined the effects of Y-320 on protein levels using the SYPRO Ruby protein stain. SYPRO Ruby, a fluorescent dye that labels positively charged amino acids and therefore most classes of proteins in the cell, was used to quantify total cellular protein by automated fluorescence microscopy. HDQ-P1 cells were exposed to G418, Y-320, or their combination for 48 h and were fixed and stained with SYPRO Ruby and with a DNA dye to identify cells. Compared to untreated cells, 200 μM G418 alone did not significantly affect cellular protein levels (Fig 3A). Unexpectedly, Y-320 increased cellular protein levels in a concentration-dependent manner, reaching 2.9-fold at 2 μM (Fig 3A). The combination of 2 μM Y-320 and 200 μM G418 also increased cellular protein, to a slightly lower level than Y-320 alone (Fig 3A).

To substantiate the increased protein level observed in Y-320-treated cells and determine whether it reflected higher protein synthesis activity, we assessed the incorporation of puromycin into nascent polypeptide chains [23]. Puromycin is a Tyr-tRNA mimetic that is incorporated into nascent chains and is commonly used to measure cellular protein synthesis. HDQ-P1 cells were exposed to G418 or Y-320, alone or in combination, for 48 h. The cells were then pulsed with puromycin for 10 min, fixed, and labeled with anti-puromycin antibody. We observed that 2 μM Y-320 alone or in combination with G418 increased puromycin incorporation 3- and 2-fold, respectively (Fig 3B). To verify that the effect of Y-320 on protein synthesis was not exclusive to one cell line, we also tested protein synthesis in H1299-R213X cells and WT fibroblasts treated with Y-320 alone for 24 h. Y-320 significantly increased protein synthesis in both cell lines (S6 Fig). These results indicate that Y-320 increases protein synthesis activity and total cellular protein levels.

The enhancement of protein synthesis by Y-320 could reflect higher metabolic demand imposed by increased cell proliferation, a feature seen in cancer cells [24,25]. To test this possibility, we exposed HDQ-P1 cells to Y-320 for 48 h and measured cell numbers. Y-320 caused a 10% to 20% reduction in cell number (S7 Fig). Additionally, increasing concentrations of Y-320 in the presence of G418 reduced HDQ-P1 cell viability up to 19% and 35% after 24 h and 48 h, respectively, as measured using the (3-[4,5-Dimethylthiazol-2-yl]-2,5-diphenyl-tetrazolium bromide (MTT) assay (S8 Fig). Therefore, enhanced protein synthesis by Y-320 was not a consequence of increased cell proliferation.

We reasoned that if a causal relationship exists between the increased protein synthesis brought about by Y-320 and its readthrough enhancement, then this correlation should hold at the single cell level. In other words, within a population of treated cells, individual cells showing higher PTC readthrough should also show higher protein synthesis. We carried out translation elongation experiments in which HDQ-P1 cells were exposed to drugs for 48 h, at which point harringtonine was added to block translation initiation, together with puromycin for 10 min. The cells were then fixed, labeled with anti-puromycin and anti-p53 antibodies, and the immunofluorescence signals were measured in approximately 2,000 individual cells per treatment using automated microscopy. The data were graphed as “lineup” plots with the cells showing lowest to highest puromycin incorporation ranked from left to right on the *x* axis and corresponding p53 levels on the *y* axis (Fig 3C). Most untreated cells displayed similar puromycin incorporation, with a minority showing a distinctly lower or higher levels. p53 levels were low in most cells, corresponding to background immunofluorescence, except for a trend toward higher p53 signal in cells showing higher puromycin incorporation (Fig 3C). In cells exposed to G418 alone, p53 levels were slightly higher in many cells (Fig 3C) and there was a weak positive linear correlation between p53 levels and translation elongation which was not different from that of untreated cells (Spearman coefficient (r) = 0.36). Most of the cells exposed to 1 μM Y-320 together with G418 had higher p53 levels, with a moderate positive correlation (r = 0.51) with translation elongation. In cells exposed to 2 μM Y-320 and G418, there was a strong positive correlation (r = 0.76) between p53 readthrough and translation elongation, with most cells showing high puromycin incorporation also showing high p53 signal (Fig 3C). Although correlative, these results are consistent with a causal relationship between increased protein synthesis and increased PTC readthrough.

### Y-320 increases ribosome biogenesis

**Fig 4:**
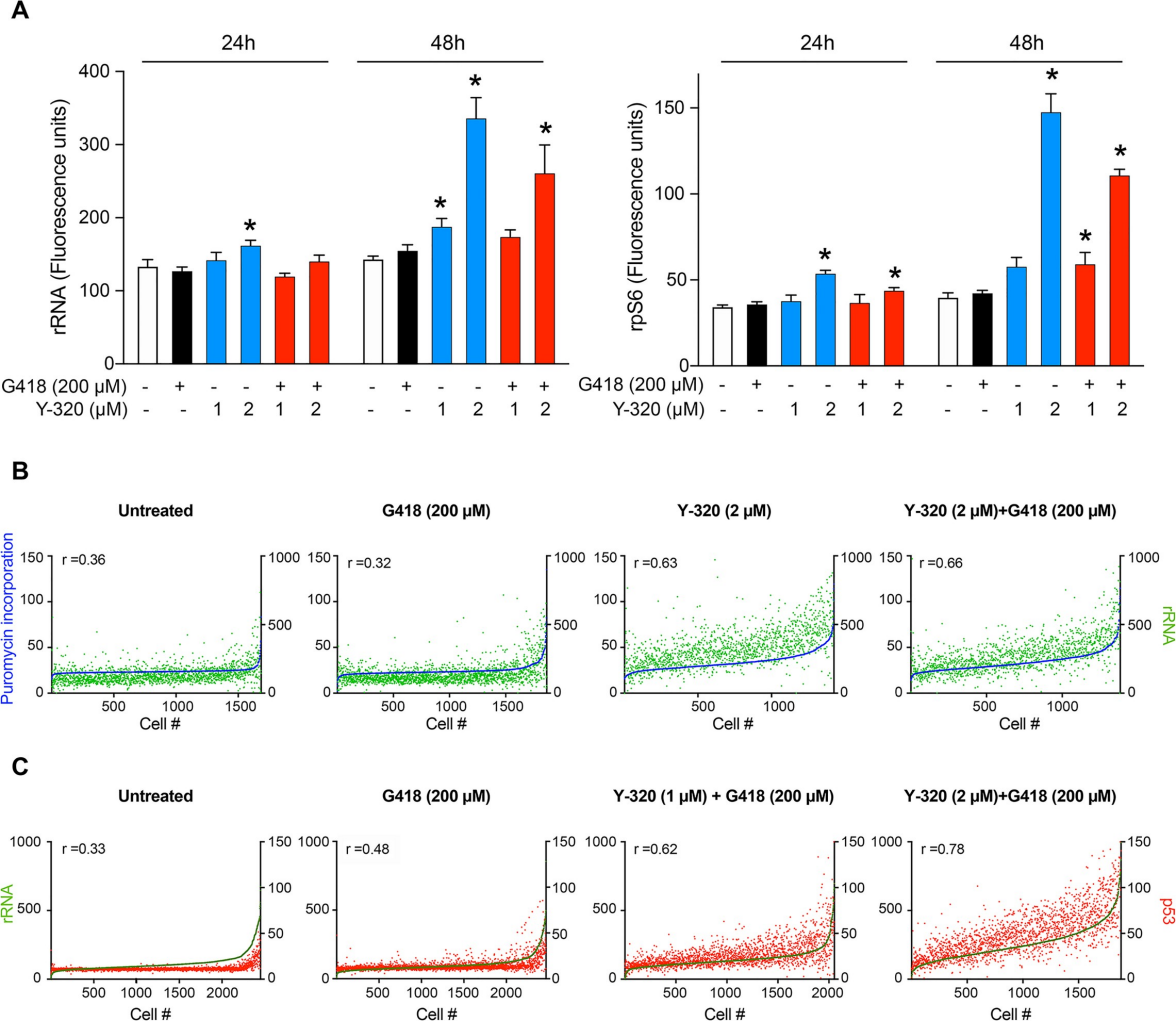
Stimulation of ribosome biogenesis by Y-320 and correlation with protein synthesis and p53 readthrough at the single cell level. (A) Ribosome biogenesis in HDQ-P1 cells. HDQ-P1 cells treated with the indicated compounds for 24 h and 48 h were fluorescently labeled using antibodies against rRNA and rpS6. Asterisks indicate values significantly different between treated and untreated cells (*p*-value < 0.05; mean ± SD; *n =* 4). (B) Quantitation of puromycin and rRNA in single cells. HDQ-P1 cells treated with the indicated compounds were exposed to 2 μg/ml harringtonine and 10 μg/ml puromycin for 10 min. Antibody staining was carried out using anti-puromycin AF488 and anti-rRNA AF647 antibodies. The graphs are representative of each condition (*n* = 4). Green dots (right *y* axis) represent rRNA expression and blue dots (left *y* axis) show puromycin labeling. Spearman coefficients (r) are shown in the graphs. (C) Quantitation of rRNA and p53 in single cells. HDQ-P1 cells treated with the indicated compounds for 48 h were fluorescently labeled using anti-rRNA AF647 and anti-p53 antibodies. The graphs are representative of each condition (*n* = 4). Red dots (right *y* axis) represent nuclear p53 expression and green dots (left *y* axis) show rRNA expression. Spearman coefficients (r) are shown in the graphs. The numerical data underlying the plots can be found in S2 Data.

The increased translation and cellular protein levels observed in cells exposed to Y-320 might reflect a higher cellular ribosomal content. To determine whether Y-320 enhanced ribosome biogenesis, we examined the expression of 2 ribosomal markers, the S40 ribosomal subunit protein rpS6 and ribosomal RNA (rRNA). HDQ-P1 cells were exposed to Y-320, G418, or combination for 24 h and 48 h, fixed and labeled with antibodies against rpS6 and rRNA. G418 alone did not affect the levels of rRNA or rpS6 (Fig 4A). However, 2 μM Y-320 alone and combined with G418 increased levels of rRNA (2.4-fold and 1.8-fold, respectively) and rpS6 (3.7-fold and 2.8-fold, respectively) relative to untreated cells at 48 h (Fig 4A). At 24 h, 2 μM Y-320 alone also increased levels of rRNA (1.2-fold) and rpS6 (1.6-fold) compared to controls. These results indicate that Y-320 stimulates ribosome biogenesis.

To determine whether the increase in ribosome biogenesis by Y-320 was associated with a higher rate of translation elongation, we used the SunRiSE run-off technique [23], whereby translation initiation is blocked with harringtonine and a puromycin pulse is applied at different times. Puromycin incorporated into elongating polypeptide chains was measured quantitatively in individual cells by automated fluorescence microscopy with an anti-puromycin antibody. Y-320 increased the level of puromycin incorporation per cell as shown by a higher *y* axis intercept (S9 Fig) confirming the higher levels of protein synthesis described above. Y-320 caused a small steepening of the negative slope, indicating a trend toward increased translation rate, but this effect was not statistically significant, indicating Y-320 had a minimal effect on the translation elongation rate. Additionally, Y-320 did not affect the levels of eIF4G or the phosphorylation of eIF4B at Ser 422, two proteins involved in control of translation initiation (S10 Fig).

Next, we explored the correlation between protein synthesis and ribosome biogenesis at the single cell level by exposing HDQ-P1 cells to drugs, incubating with harringtonine and puromycin, and staining the cells with anti-puromycin and anti-rRNA antibodies.

Exposure to Y-320 without or with G418 increased rRNA levels in the vast majority of cells (Fig 4B) and a positive correlation between rRNA levels and translation elongation was observed (r = 0.63 and r = 0.66, respectively), consistent with larger numbers of ribosomes leading to increased protein translation.

To determine whether there was any connection between ribosome biogenesis and potentiation of readthrough by Y-320, we also examined rRNA expression versus p53 expression at the single cell level. We treated HDQ-P1 cells with G418 or its combination with Y-320 for 48 h and performed immunofluorescence imaging of cells double-stained for p53 and rRNA (Fig 4C). Upon exposure to the combination of Y-320 and G418, a strong positive correlation was observed between higher levels of rRNA and higher p53 levels (r = 0.78 in 2 μM Y-320 + 200 μM G418). Together, the results presented in Figs 3 and 4 indicate that ribosome biogenesis, cellular protein levels, and translation elongation all positively correlate with PTC readthrough after combination treatment with Y-320 and G418. These results are consistent with higher numbers of ribosomes enabling higher levels of protein synthesis and higher levels of PTC readthrough.

### Y-320 stimulates protein synthesis independently of mTOR signaling

**Fig 5:**
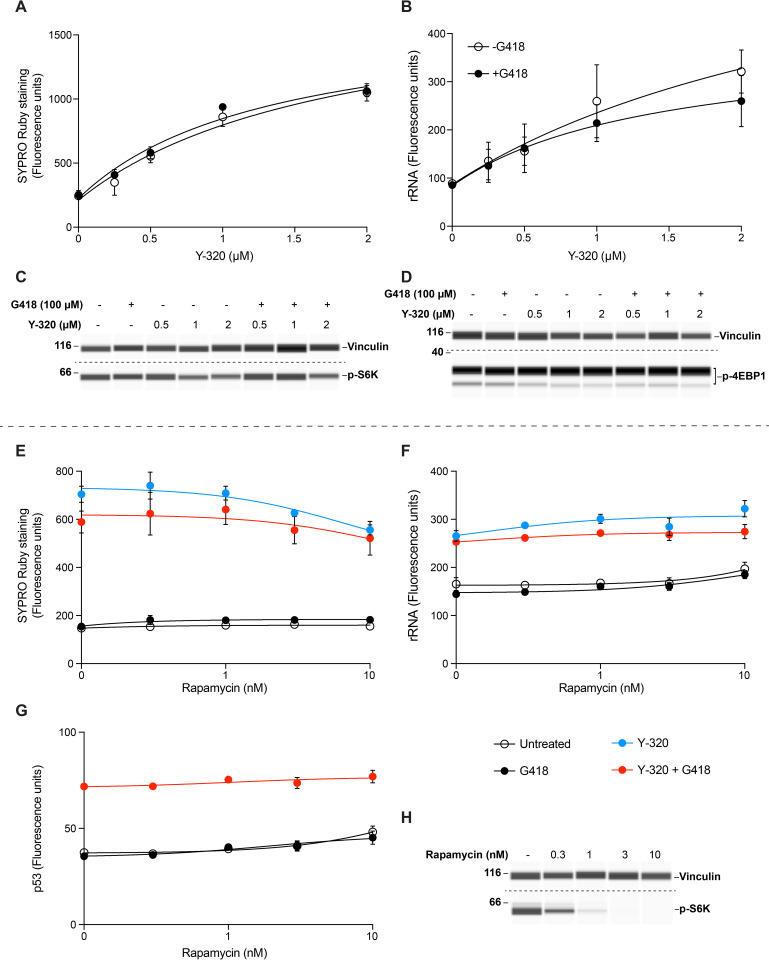
Y-320 stimulates protein synthesis independently of mTOR signaling. (A–D) Effect of Y-320 on *Tsc2*^*−/−*^ cells. Cells were exposed to 100 μM G418 alone or in combination with the indicated concentrations of Y-320 for 48 h. They were then stained with SYPRO Ruby (A) or fluorescently labeled using rRNA antibody (B) (*n =* 4 for A and B). Cells were lysed and equal amounts of protein were subjected to automated capillary electrophoresis western analysis. Shown are pseudo blots of chemiluminescence of bound p-S6K (C) and p-4EBP1 (D) antibodies. Vinculin was used as the loading control. (E–H) Effect of rapamycin in HDQ-P1 cells. HDQ-P1 cells were exposed to different concentrations of rapamycin alone or in combination with the indicated compounds for 48 h. Cells were then stained with SYPRO Ruby (E) or were fluorescently labeled using antibodies against rRNA (F) or p53 (G) (*n* = 3 for E–G). (H) HDQ-P1 cells exposed to different concentrations of rapamycin for 48 h were lysed, and equal amounts of protein were subjected to automated capillary electrophoresis western analysis. Shown are pseudo blots of chemiluminescence of bound p-S6K antibody, and vinculin, used as the loading control. Uncropped images of automated capillary electrophoresis western analyses are provided in S1 Raw Images, and numerical data underlying the plots can be found in S3 Data. mTOR, mammalian target of rapamycin; p-S6K, phospho-S6K (Thr389); p-4EBP1, phospho-4EBP1 (Thr 37/46).

Having established that Y-320 increases protein synthesis and ribosome biogenesis, we sought to determine the relationship of these effects with mammalian target of rapamycin (mTOR) signaling, a pathway known to control protein synthesis and ribosome biogenesis [26,27]. The tuberous sclerosis complex genes *TSC1* and *TSC2* are negative regulators of mTOR signaling [28]. We examined the effect of Y-320 on cellular protein and ribosomal RNA in immortalized *Tsc2*^*−/−*^ mouse embryo fibroblasts, which feature constitutively high mTOR activity [28,29]. *Tsc2*^*−/−*^ cells were exposed to Y-320 without or with G418 for 48 h, fixed and probed with SYPRO Ruby stain and anti-rRNA antibody. Y-320 increased levels of cellular protein and rRNA up to 4.3-fold and 3.6-fold, respectively, compared to controls (Fig 5A and 5B). Activation of mTOR signaling leads to phosphorylation of downstream proteins ribosomal S6 kinase (S6K) and translation initiation factor 4E-binding protein 1 (4EBP1) [26,30]. To determine whether Y-320 affected mTOR activity, lysates from *Tsc2*^*−/−*^ cells exposed to compounds as above were subjected to automated capillary electrophoresis western analysis to quantitate the levels of phosphorylated S6K and 4EBP-1. Y-320 caused a small decrease in phospho-S6K but it did not change the phosphorylation of 4EBP1 (Fig 5C and 5D). These results indicate that Y-320 can increase cellular protein levels and ribosome biogenesis even in the presence of highly active mTOR signaling.

To determine the effect of mTOR inhibition on cellular protein, ribosome biogenesis, and PTC readthrough, we used rapamycin, a potent inhibitor of mTOR signaling [26,31]. HDQ-P1 cells maintained in normal culture medium, which is rich in glucose, serum, and amino acids, were exposed to rapamycin at concentrations between 0.3 and 10 nM for 48 h and phosphorylation of S6K was assessed. Rapamycin reduced S6K phosphorylation in a concentration-dependent manner with strong inhibition at 3 nM and above (Fig 5H), as previously reported in other cell lines [26,32]. Next, we exposed HDQ-P1 cells to Y-320 without or with G418, together with different concentrations of rapamycin for 48 h, fixed and stained them with SYPRO Ruby or fluorescently labeled them using the antibodies against rRNA and p53. We observed that rapamycin by itself did not affect the overall protein levels, as previously described [26] nor did it inhibit the effects of Y-320 on cellular protein, rRNA, or p53 levels (Fig 5E–5G), except for a minor reduction in SYPRO Ruby staining at 10 nM rapamycin. These results indicate that inhibition of mTOR signaling is not sufficient to prevent Y-320 from increasing protein levels, ribosome biogenesis, or PTC readthrough and that the effects of Y-320 are not mediated by mTOR signaling.

### Effect of Y-320 on translation fidelity

**Fig 6:**
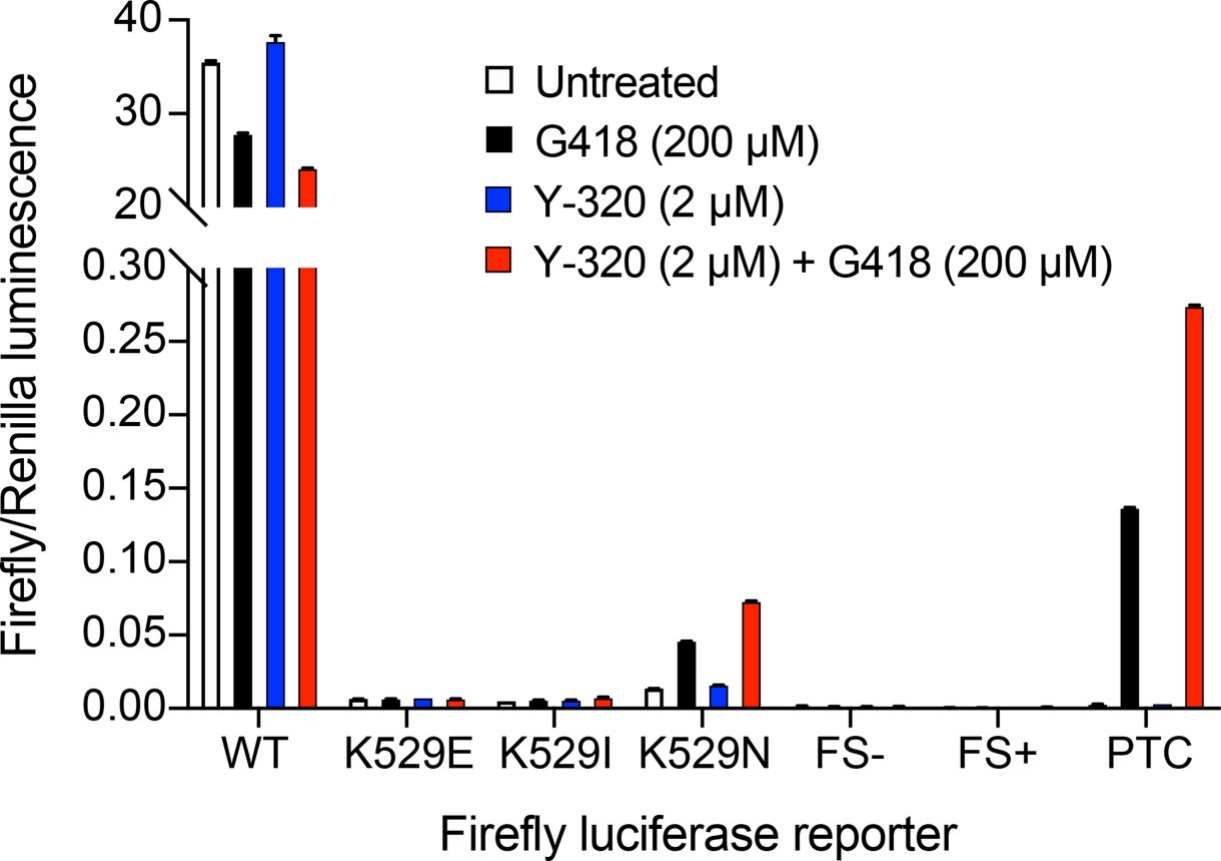
Effect of Y-320 on translation. H1299 cells transfected with WT Renilla and WT or mutated firefly luciferase plasmids were treated with the indicated compounds for 48 h. After cell lysis, mutated firefly luminescence was measured and normalized to that of Renilla luciferase (mean ± SD; *n =* 3). The positive control (WT-Luc) ratio was several orders of magnitude higher and was cropped on the graph. The mutant reporters K529E, K529I, and K529N measured misincorporation into the first, second, and third codon positions, respectively. The mutant FS+ contained a positive frameshift mutation by nucleotide insertion at position 81, whereas FS− mutant contained a negative frameshift mutation by nucleotide deletion at position 81. The PTC reporter contained a premature stop codon at amino acid 81. The numerical data underlying the plot can be found in S4 Data. FS, frameshift; PTC, premature termination codon; WT-Luc, wild-type luciferase.

Given the surprising result that Y-320 increased cellular protein synthesis, we wondered to what extent this process might be dysfunctional. We assessed translation fidelity during Y-320 treatment using a set of firefly luciferase mutant reporters in H1299 cells [33]. In these reporters, lysine 529 is mutated, leading to defective luciferase activity, and misincorporation of lysine at the mutated site results in restoration of luciferase activity. The mutations consisted of nucleotide substitution at the first (K529E), second (K529I), and third (K529N) codon positions, a premature stop codon, and 2 frameshift mutations (FS+ and FS−). Cells transfected with the WT luciferase construct showed a high level of luciferase activity which was strongly diminished in the cells transfected with the mutant reporters (Fig 6). G418 alone had no effect on the frameshift mutations or on missense mutations K529E and K529I but it increased amino acid misincorporation at K529N and it induced readthrough at the PTC. Y-320 alone had no effect on any of the mutants, indicating no observable effect on translation fidelity. The combination of G418 and Y-320 had no observable effect on missense mutations K529E, K529I, or the frameshift mutants. However, the combination of Y-320 and G418 increased PTC readthrough by G418, as expected, and it also increased misincorporation at K529N compared to G418 alone (*p*-value < 0.0001). These data indicate that the increased protein translation that takes place during exposure to Y-320 alone maintains a high level of translation fidelity. However, in addition to increasing G418-induced PTC readthrough, Y-320 also amplifies the effect of G418 on amino acid misincorporation at the third codon position.

### Y-320 up-regulates CXC chemokine expression

Little is known about the mechanism of action of Y-320, other than it inhibited IL-17 production by murine CD4 T cells stimulated with a cocktail of IL-15, CXCL12, and anti-CD3 monoclonal antibody (mAb) [20]. Seeing no obvious link between this pharmacological effect and stimulation of ribosome biogenesis, protein synthesis, and PTC readthrough, we decided to examine the effect of Y-320 on the transcriptome. RNA samples isolated from HDQ-P1 cells treated with 1 μM Y-320 for 48 h were compared to those of untreated cells, and the RNA sequencing (RNA-seq) dataset was analyzed for differentially expressed genes.

**Fig 7:**
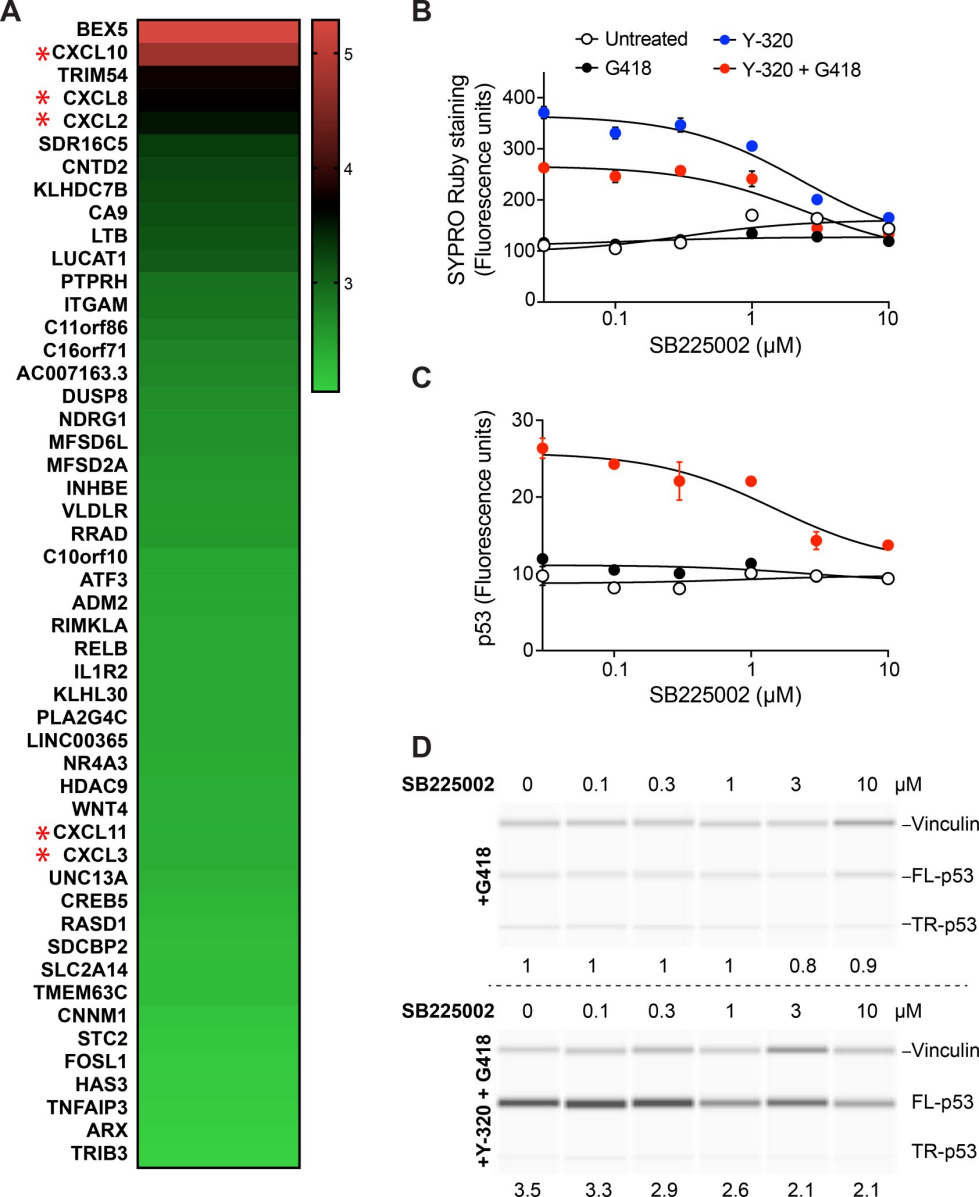
Up-regulation of CXC chemokines by Y-320 and effect of SB225002 on protein levels and PTC readthrough. (A) Heat map of RNA-seq data of the 50 genes with highest RNA expression level in HDQ-P1 cells treated with 1 μM Y-320 for 48 h relative to untreated cells. CXCL genes are marked with red asterisks. The underlying data for the RNA-seq heat map can be found in the GEO database (accession number GSE152142). Key bar = log_2_ fold change. (B) SYPRO Ruby protein staining. HDQ-P1 cells treated with 200 μM G418, 2 μM Y-320, 200 μM G418 + 2 μM Y-320, and different concentrations of SB225002 (Selleck Chemicals S7651) for 48 h were stained using SYPRO Ruby. Data were collected by automated fluorescence microscopy (mean ± SD; *n =* 4). (C, D) Effect of SB225002 on PTC readthrough. HDQ-P1 cells were treated as in B, and readthrough was analyzed by automated fluorescence microscopy (mean ± SD; *n* = 3) (C) and p53 automated capillary electrophoresis western analysis (D). In panel D, the area under the full-length p53 peaks was first normalized to the vinculin loading control and then divided to that of G418 alone to provide lane-to-lane comparison. These numbers are displayed under the lanes. Uncropped images of automated capillary electrophoresis western analyses are provided in S1 Raw Images, and numerical data underlying the plots can be found in S5 Data. FL-p53, full-length p53; PTC, premature termination codon; RNA-seq, RNA sequencing; TR-p53, truncated p53.

Y-320 up-regulated the expression of several genes (Fig 7A). Considering that 3 chemokines were among the 5 most up-regulated genes and the previously reported immunomodulatory activity of Y-320 [34], we decided to focus on these genes. CXC chemokine ligands CXCL10, CXCL8, and CXCL2 showed log_2_ fold increase >3, and CXCL11 and CXCL3 showed log_2_ fold increase >2 (Fig 7A). CXCL1 and CXCL16 were also up-regulated by Y-320 although to a lesser degree (log_2_ fold = 1.42 and 0.47, respectively). To validate the RNA-seq data, we measured the mRNA levels of these CXCL chemokines in untreated and Y-320 treated cells using qPCR. The results were very similar to those of RNA-seq with *CXCL10* mRNA showing the highest expression compared to control (S11 Fig). CXC chemokines are small secreted proteins that have been attributed several roles including chemotaxis in cells expressing receptors for these chemokines [35]. CXCL10, CXCL8, CXCL2, and CXCL3 can also induce cell growth and proliferation and are putative autocrine or paracrine growth factors in cancer [36–38]. CXCL8, CXCL2, CXCL3, and CXCL1 act through a common receptor, CXCR2 [39], which showed no up-regulation in our RNA-seq data.

SB225002 is a non-peptide antagonist of CXCR2 which has been shown to inhibit chemokine-induced neutrophil migration [40,41]. To investigate whether the mechanism of action of Y-320 involves signaling through CXCR2, we examined the effect of SB225002 on cellular protein levels and PTC readthrough. HDQ-P1 cells were exposed to Y-320, G418, or combination of the two, together with different concentrations of SB225002 for 48 h, fixed and probed with SYPRO Ruby stain and anti-p53 antibody. SB225002 did not affect protein levels in untreated cells or cells treated with G418 alone (Fig 7B). The increase in cellular protein levels elicited by Y-320 alone and Y-320 combined with G418 was reduced by SB225002 in a concentration-dependent manner (Fig 7B). SB225002 also considerably decreased in a concentration-dependent manner PTC readthrough elicited by the combination of G418 and Y-320, both in the immunofluorescence assay (Fig 7C) and by western analysis (Fig 7D). These results indicate that the effects of Y-320 on increased protein synthesis and PTC readthrough are mediated in large part by an autocrine mechanism involving up-regulation of chemokine expression and secretion and increased signaling through a chemokine receptor.

## Discussion

Aminoglycosides suffer several limitations as potential PTC readthrough treatments for rare genetic disorders. At the organismal level, they are known to cause oto- and nephro-toxicity via preferential accumulation and retention in cochlear cells and proximal tubule cells [42–44]. At the cellular level, their toxicity has been linked to damage to the lysosomal membrane following uptake by endocytosis and accumulation in this compartment [45–47], as well as inhibition of mitochondrial protein synthesis [12,15]. Here, we illustrate a further limitation: inhibition of cytosolic protein synthesis at the G418 concentrations that are required for readthrough. As a result, the maximum levels of PTC readthrough achievable by G418 are low, even in a cell-free translation assay, where the other factors described above are not at play. Whether inhibition of protein synthesis at concentrations that induce readthrough is due to higher occupancy of the decoding center or to binding to additional sites on the ribosome remains to be deteremined. It will likely be important to also consider this self-limiting process when developing improved aminoglycosides for readthrough therapy.

Here, we asked whether PTC readthrough could be increased without further inhibiting protein synthesis by combining G418 with another small molecule. We first identified Y-320 as a potent enhancer of G418-induced PTC readthrough active against nonsense mutations in different genes and patient-derived cells of different tissue origins. When used alone, Y-320 did not increase R213X *TP53* mRNA levels, indicating it is not an NMD inhibitor. The combination of Y-320 and G418 increased R213X *TP53* mRNA to a higher level than G418 alone, which is likely a consequence of increased readthrough by the combination, as is the higher level of truncated p53 [9,16]. In the particular case of p53, which acts as a tetramer, the presence of truncated p53 has potential to inhibit p53 function by a dominant-negative mechanism [48,49]. The levels of full-length products induced by readthrough in DMD and JEB cells were very low compared to those of WT myoblasts and keratinocytes. However, considering the long half-lives (>1 month) of dystrophin and collagen XVII, repeated administration of readthrough combinations in an animal model could potentially cause accumulation of higher levels of readthrough protein over time.

While investigating whether combining G418 with Y-320 would negatively affect protein synthesis, we made the surprising observation that Y-320 actually enhanced protein synthesis. This finding was unanticipated as cells were maintained in culture medium rich in glucose, serum, and amino acids, conditions that presumably favored robust rates of growth and protein synthesis. The unanticipated effect of Y-320 on protein synthesis was verified using a cellular protein dye and puromycin incorporation, as well as a protein synthesis click chemistry approach (S6 Fig). Moreover, cells exposed to Y-320 showed higher levels of ribosomal markers rRNA and ribosomal protein S6, consistent with stimulation of ribosome biogenesis underlying the increased cellular protein levels and synthesis. It is noteworthy that CDX5-288 [16] and CDX6-180, two structurally distinct readthrough enhancers recently identified in our laboratory, showed no stimulation of ribosome biogenesis (S12 Fig).

To our knowledge, increased protein synthesis activity has not previously been linked to enhancement of PTC readthrough by an aminoglycoside. We were interested to determine whether a causal relationship existed between these 2 processes. This is not an easy question to address as protein synthesis is fundamentally linked to essentially all cellular activities, and it is not suited to gene down-regulation or overexpression approaches often used to validate drug targets. We first elected to verify whether the correlation observed between increased protein synthesis, increased ribosomal markers, and increased PTC readthrough observed at the cell population level also held at the cellular level, reasoning that a lack of association at the individual cell level would indicate unrelated effects. A strong correlation was observed between increased rRNA levels and puromycin incorporation, which was not surprising and indicated the additional ribosomes produced during incubation with Y-320 were functional. In individual cells exposed to G418 and 2 μM Y-320, we also observed strong positive correlations between puromycin incorporation and PTC readthrough (r = 0.76) and between rRNA levels and PTC readthrough (r = 0.78), which encouraged us to further investigate this relation.

Y-320 stimulated both ribosome biogenesis and protein synthesis but it did not significantly increase the rate of translation elongation. We asked whether the ribosomes produced during exposure to Y-320 might show lower translation fidelity, which would increase the likelihood of incorporating an amino acid at a PTC. However, experiments with highly sensitive reporter plasmids indicated that Y-320 alone does not cause frameshifting or increase misincorporation at sense codons or at a PTC. It is only in the presence of G418 that increased PTC readthrough was observed, in agreement with our results on readthrough of endogenous genes. The combination also increased incorporation at the third codon position (K to N) (Fig 6), where misreading most often occurs because interaction at the cognate base is weaker than at the first and second positions [50,51].

Y-320 was discovered in a phenotypic cell-based screen as a compound that inhibited IL-17 production by murine CD4 T cells stimulated with a cocktail of IL-15, CXCL12, and anti-CD3 mAb [20,34]. Y-320 also ameliorated type II collagen-induced arthritis in mice and monkeys and reduced IL-17 mRNA expression in arthritic joints in mice [20]. However, a direct target for Y-320 and its cellular mechanism of action have not been described. A recent study has shown that Y-320 can also inhibit the P-glycoprotein efflux pump and sensitize multidrug resistant tumors to chemotherapy [52]. We examined whether the effect of Y-320 was dependent on mTOR, a major regulator of protein synthesis and ribosome biogenesis. Experiments with *Tsc2*^*−/−*^ cells expressing high levels of mTOR activity and with the mTOR inhibitor rapamycin indicated no involvement of mTOR. To further probe the mechanism of action of Y-320, we determined its effects on gene expression in HDQ-P1 cells. Our RNA-seq data showed Y-320 strongly up-regulated the expression of chemokines CXCL10, CXCL8, CXCL2, CXCL11, and CXCL3, and moderately up-regulated CXCL1 and CXCL16. Chemokines and their receptors are produced by many cells including fibroblasts, immune cells, and cancer cells. Chemokines have well established roles in homeostasis and controlling cell migration during organ development as well as leukocyte activation and trafficking during immune responses [35,53,54]. Chemokines also play roles in the biology of nonimmune cells. Chemokines expressed by cancer cells and stromal cells can stimulate tumor growth, progression and metastasis [35,55,56], and chemokines have been described as putative autocrine or paracrine growth factors in cancer [36–38]. To our knowledge, the effect of CXCL chemokines on ribosome biogenesis and protein synthesis has not been investigated, but proinflammatory cytokines have previously been shown to increase the global translation rate in bronchial epithelial cells [57].

CXCL8, CXCL2, CXCL3, and CXCL1 exert their effects through the receptor CXCR2 [39]. Here, the CXCR2 antagonist SB225002 reduced the increase in protein levels and PTC readthrough caused by Y-320 and G418 while having no effect on basal protein synthesis in cells not exposed to the readthrough compounds. This key experiment suggests a model whereby Y-320, at least in part, exerted these effects in an autocrine manner through up-regulating the expression and secretion of chemokines that then bound to cell surface CXCR2 to induce an intracellular signal that increased protein synthesis and PTC readthrough. One implication of this mechanism is that pro-inflammatory signals might generally enhance PTC readthrough by an aminoglycoside. This may be relevant to the treatment of recessive dystrophic EB (RDEB) in patients with nonsense mutations in the *COL7A1* gene. It has been observed that topical or intradermal application of gentamicin on open skin wounds of RDEB patients could strongly induce readthrough and the expression of full-length COL7 protein, up to 165% of that of normal human skin [58]. This level of expression is much higher than what can be achieved in vitro in cultured keratinocytes [59]. Based on our observations, it is tempting to speculate that in those patients, readthrough by gentamicin might have been enhanced by the inflammatory environment at the skin lesions.

In conclusion, the findings of this study indicate that the self-limiting nature of PTC readthrough by an aminoglycoside like G418 can be overcome by Y-320, a compound that increases ribosome biogenesis and protein synthesis. The data support an unusual mechanism of action involving increased protein synthesis mediated by an autocrine pathway involving chemokine signaling.

## Materials and methods

### PTC readthrough screening assay

One day after seeding HDQ-P1 cells at 4,500 cells per well in 96-well plates, the cell culture medium was replaced with fresh medium containing 50 μM G418. A total of 2,658 compounds (Selleckchem 96-well-Z202687-100μl-L1700) were added to each well using a Biorobotics Biogrid II robot equipped with a 0.4-mm diameter 96-pin tool. After 72 h, the medium was removed and cells were rinsed with phosphate-buffered saline (PBS). Cells were then fixed, permeabilized, and stained for nuclei using 3% paraformaldehyde, 0.3% Triton X-100, and 1.5 μg/ ml Hoechst 33323 in PBS for 20 min. Following PBS wash, cells were kept in blocking buffer (3% bovine serum albumin (BSA) in PBS) for 2 h at room temperature. Cells were incubated with mouse anti-p53 (1:1,000, DO-1, Santa Cruz sc-126) in blocking buffer for 1.5 h, rinsed with PBS, and labeled with goat anti-mouse Alexa 488 secondary antibody (1:1,000; Thermo Fisher Scientific) for 1.5 h at room temperature. Cells were then thoroughly washed with PBS, covered with a black membrane, and stored at 4°C until imaging. Imaging was performed using a Cellomics ArrayScan VTI automated fluorescence microscope and 12 fields per well at 20× magnification were captured. The average nuclear p53 immunofluorescence intensity was used for statistical analysis.

### Cell lines

The HDQ-P1 cell line was purchased from the German Collection of Microorganisms and Cell Cultures (DSMZ, Germany). HSK001 myoblasts, derived from a patient with DMD harboring nonsense mutation in the *DMD* gene, were generated as previously described [16]. NCI-H1299 cells were purchased from the American Type Culture Collection (ATCC, United States of America). Immortalized JEB01 keratinocytes from a patient with JEB carrying homozygous nonsense mutations in the *COL17A1* gene were provided by Cristina Has, University of Freiburg. HaCaT cells, a spontaneously transformed keratinocyte cell line from adult human skin were used as controls. *Tsc2*^*−/−*^ *TP53*^*−/−*^ mouse embryonic fibroblasts (MEF) [29] were obtained from David Kwiatkowski, Harvard Medical School. HDQ-P1 cells and *Tsc2*^*−/−*^ *TP53*^*−/−*^ MEFs were cultured in high glucose DMEM supplemented with 10% (vol/vol) fetal bovine serum (FBS) and 1% antibiotic–antimycotic (Gibco/Thermo Fisher Scientific). H1299 cells were cultured in RPMI-1640 medium supplemented with 10% FBS and 1% antibiotic–antimycotic. The cells were always maintained at 70% to 80% confluency at the time of the assays. For DMD experiments, human myoblasts were cultured in Skeletal Muscle Cell Growth Medium (PromoCell C-23060) supplemented with 20% (vol/vol) FBS and 1% antibiotic–antimycotic. Myoblasts were differentiated into myotubes at full confluency in differentiation medium consisting of high glucose DMEM (Gibco/Thermo Fisher) supplemented with 10 μg/ml insulin (Millipore Sigma 91077) and 1% antibiotic–antimycotic. Keratinocytes were cultured in defined keratinocyte serum-free medium (K-SFM) supplemented with defined K-SFM growth supplement (Gibco/Thermo Fisher Scientific) and 1% antibiotic–antimycotic. All cell lines were cultured at 37°C and 5% CO_2_, and medium was replaced every 2 to 3 days.

### Automated capillary electrophoresis western analysis

The levels of vinculin, GAPDH, p53, and dystrophin were measured as previously described with minor modifications [16]. Briefly, cell lysates were precleared by centrifugation at 15,000*g* for 10 min at 4°C. Mixture of cell lysates (1 mg/ml) and the fluorescent master mix were heated at 95°C. The samples, blocking reagent, wash buffer, primary antibody, secondary antibody, and chemiluminescent substrate were dispensed into the microplate provided by the manufacturer (ProteinSimple WES, United States of America). The electrophoretic separation and immunodetection were carried out automatically using default settings. Mouse anti-p53 antibody (1:400, DO-1, Santa Cruz sc-126), mouse anti-vinculin antibody (1:600 for HDQ-P1 and 1:1,000 for H1299-R213X and *Tsc2*^*−/−*^ cells, R&D Systems MAB6896), rabbit anti-phospho-p70 S6K (Thr389) (1:50, Cell Signaling Technology 9205), rabbit anti-phospho-4EBP1 (Thr37/46) (1:200, Cell Signaling Technology 9459), rabbit anti-Collagen XVII (1:25, Abcam ab184996), rabbit anti-beta-actin (1:10,000, Novus Biologicals), rabbit anti-Dystrophin (1:400, Abcam ab15277), rabbit anti-eIF4G (1:100, Cell Signaling Technology 2498), and rabbit anti-phospho-eIF4B (Ser422) (1:150, Cell Signaling Technology 3591) were used. Data were presented as pseudo blots and analyzed using the built-in Compass software (ProteinSimple). The full-length p53 peak intensities (area under the curve) were normalized to that of the vinculin peak, used as a loading control.

### SDS-PAGE and immunoblotting

One day after seeding human JEB01 keratinocytes at 2 × 10^5^ cells/2 ml/well in 6-well plates, the cell culture medium was replaced with fresh medium containing G418 alone or G418 + Y-320. After 72 h, cells were lysed and 15 μg total protein from each lysate was separated on a 6% polyacrylamide gel. Gels were electrotransferred onto a nitrocellulose membrane and blocked in Tris-buffered saline containing 0.1% (v/v) Tween 20 and 5% (w/v) nonfat dry milk (TBS-T-milk). Membranes were incubated with rabbit anti-Collagen XVII (1:1,000, Abcam ab184996) overnight at 4°C. After washing with TBS-T, membranes were incubated with HRP-conjugated goat anti-rabbit secondary antibody and developed using enhanced chemiluminescence substrate (Millipore). After stripping using 0.1 N NaOH, membranes were reprobed with rabbit anti-beta-actin (1:10,000, Novus Biologicals) and detected as above. To quantify the signal, each lane was selected using the selection tool in ImageJ after which the area was plotted and measured using the “Plot Lanes” icon in ImageJ.

### SYPRO Ruby protein staining

One day after seeding HDQ-P1 cells at 5,000 cells per well in 96-well plates, the cell culture medium was replaced with fresh medium containing the indicated compounds. After 48 h, the cell monolayers were rinsed with PBS, fixed in cold 100% MeOH for 15 min, and stained with FilmTracer SYPRO Ruby biofilm matrix stain (Thermo Fisher Scientific F10318) containing 1.5 μg/ml Hoechst 33323 for 30 min at room temperature. The cells were then washed with PBS, covered with black membrane, and stored at 4°C for imaging. Imaging was performed using a Cellomics ArrayScan VTI automated fluorescence microscope, and 12 fields per well at 20× magnification were captured. The XF93-TRITC filter was used to detect SYPRO Ruby signal. The average nuclear and cytoplasmic signal intensities were used as an indicator of cellular protein level for statistical analysis. Y-320 showed no autofluorescence in this or other assays described in this study.

### Automated immunofluorescence microscopy

One day after seeding HDQ-P1 cells at 5,000 cells per well in 96-well plates, the cell culture medium was replaced with fresh medium containing the indicated compounds. After 24 h or 48 h, cell monolayers were rinsed with PBS and fixed using cold 100% MeOH for 15 min. Cells were then permeabilized and stained for nuclei using 0.1% Tween-20 and 1.5 μg/ml Hoechst 33323 in PBS for 20 min at room temperature. Cells were quickly rinsed with PBS and kept in 3% BSA in PBS blocking buffer for 1 h. After blocking, cells were incubated with mouse anti-p53 (1:1,000, DO-1, Santa Cruz sc-126), mouse anti-rRNA (1:100, Y10b, Santa Cruz sc-33678), or mouse anti-ribosomal protein S6 (1:100, C-8, Santa Cruz sc-74459) for 1 h at room temperature. Cells were then labeled with Alexa 488-conjugated goat anti-mouse (1:1,000, Thermo Fisher Scientific) secondary antibody for 30 min at room temperature. For rRNA and p53 double staining, cells were incubated with a mixture of Alexa 647-conjugated anti-rRNA (1:50, Y10b, Santa Cruz sc-33678 AF647) and mouse anti-p53 (1:1000, DO-1, Santa Cruz sc-126) for 1 h at room temperature. Cells were washed and incubated with Alexa 488-conjugated goat anti-mouse secondary antibody in 3% BSA (in PBS) for 30 min. After several washes with PBS, cells were covered with a black membrane and stored in PBS at 4°C until imaging.

For puromycin labeling, after indicated treatments, HDQ-P1 cells in 96-well plates were exposed to 10 μg/ml puromycin (Millipore Sigma P7255) with or without 2 μg/ml harringtonine (Santa Cruz sc-204771). For the SunRiSE experiment, cells were exposed to harringtonine (5 μg/ml) for different times prior to incubation with puromycin (10 μg/ml) at 37°C and 5% CO_2_ for 10 min. The cell monolayers were then rinsed with cold PBS, fixed in cold 100% MeOH for 15 min, and permeabilized and stained for nuclei using 0.1% Tween-20 and 1.5 μg/ml Hoechst 33323 in PBS. The cells were incubated in blocking buffer (3% BSA in PBS) for 1 h and labeled with Alexa 488-conjugated anti-puromycin antibody (1:400, Millipore Sigma MABE343-AF488) for 1 h at room temperature. Cells were then thoroughly washed with PBS, covered with a black membrane, and stored at 4°C until imaging. For puromycin and p53 or rRNA double staining, after blocking, cells were incubated with the mixture of Alexa 488-conjugated anti-puromycin (1:400) and Alexa 594-conjugated anti-p53 (1:100, DO-1, Santa Cruz sc-126 AF594) or Alexa 647-conjugated anti-rRNA (1:50) antibodies for 1 h at room temperature. After several washes with PBS, cells were covered with a black membrane and stored in PBS at 4°C until imaging.

For all immunofluorescence experiments, imaging was performed using a Cellomics ArrayScan VTI automated fluorescence microscope, and 12 fields per well at 20× magnification were captured. The average nuclear immunofluorescence intensity was measured and used as an indicator of p53 expression. The average nuclear and cytoplasmic immunofluorescence intensities were measured and used as indicators of puromycin labeling, rRNA and rpS6 expression in data analysis.

### Click-iT chemistry

The Click-iT HPG Alexa Fluor 488 Protein Synthesis Assay Kit (Thermo Fisher Scientific 10428) was used as instructed by the manufacturer. Briefly, following the treatments, WT fibroblast monolayers were rinsed with PBS and incubated in DMEM without methionine at 37°C and 5% CO2 for 30 min. Click-iT L-homopropargylglycine (HPG) reagent (1:1000) was then added to media for another 30 min. Cells were rinsed with PBS, fixed in cold 100% MeOH for 15 min, permeabilized with 0.3% Tween-20 in PBS for 20 min, and incubated in Click-iT reaction cocktail for 30 min in the dark. The cells were washed using the rinse buffer provided in the kit and stained for the nuclei using the provided nuclear stain. The cells were then washed with PBS, covered with a black membrane, and stored at 4°C until imaging. Cells incubated without HPG reagent were used for determination of background fluorescence in statistical analysis. Imaging was performed using a Cellomics ArrayScan VTI automated fluorescence microscope, and 12 fields per well at 20× magnification were captured. The average nuclear and cytoplasmic signal intensities were used as an indicator of protein synthesis for statistical analysis.

### In vitro translation

The in vitro translation assay was carried out using the 1-Step Human Coupled IVT Kit (Thermo Fisher Scientific) according to the manufacturer’s instructions and as previously described with minor modifications [16]. Briefly, 500 ng 5′-capped and poly(A) tailed p53-WT or p53-R213X mRNA was added to the Hela cell lysate-based protein expression system and incubated at 30°C for 20 min with different concentrations of G418. Samples were diluted 10-fold in water and subjected to automated electrophoresis western analysis for p53 detection.

### Cell viability assay

HDQ-P1 cells were seeded at 5,000 cells/well in 96-well plates. The next day, cells were exposed to various concentrations of Y-320 with or without 200 μM G418. After 48 h, cell viability was determined by MTT assay as previously described [60]. In brief, 25 μl of a 5-mg/ml solution of 3-(4,5-dimethylthiazol-2-yl)-2,5-diphenyltetrazolium bromide (Sigma) in PBS was added to cells in the presence of 100 μl of cell culture medium and incubated at 37°C. After 2 h, 100 μl of extraction buffer (20% sodium dodecyl sulfate dissolved in dimethylformamide/water (1:1), pH 4.7) was added, and cells were incubated at 37°C. After 4 h, the absorbance at 570 nm was measured.

### Luciferase assay

The pGL3 luciferase plasmids were generously provided by Dr. Vera Gorbunova (University of Rochester). One day after seeding H1299 cells at 2 × 10^5^ cells/well in 12-well plates, cells were transfected with 500 ng firefly reporter plasmid (WT or mutant) and 10 ng Renilla luciferase plasmid using lipofectamine 2000 (Thermo Fisher Scientific). Transfected cells were treated with the indicated compounds 24 h after transfection and were lysed 48 h later using the passive lysis buffer provided in the Dual-Luciferase assay kit (Promega). The luminescence of firefly and Renilla was measured in an automated luminometer (Fluoroskan Ascent) after addition of LARII and Stop & Glo buffers, respectively. The ratio of firely/Renilla luminescence was used in data analysis.

### RNA isolation, sequencing, and analysis

RNA of treated and untreated replicates (*n =* 3) of HDQ-P1 cells was extracted using RNeasy kit (Qiagen) following the manufacturer’s protocol. Total RNA quality and concentration was assessed using an Agilent 2100 Bioanalyzer (Agilent). Libraries for RNA-seq were generated using the Illumina Neoprep System and sequenced using the IlluminaNextseq500 (Illumina). As previously described [61], multiple analysis pipelines were applied and their results combined. The pipelines used the reference genome and transcriptome hg38 downloaded from Ensembl (www.ensembl.org). Only genes achieving the significance cut-off in at least half the pipelines were considered as significantly differentially expressed. Differential expression was determined by log_2_ fold change between control and Y-320 treated values.

### RT-PCR

Quantitative Real-Time PCR for detection of *CXCL1*, *CXCL2*, *CXCL3*, *CXCL8*, *CXCL10*, *CXCL11*, and *TP53* mRNA was performed using the ABI StepOnePlus Real-Time PCR system, as previously described [16] using the primers listed in S2 Table.

### Statistical analysis

Statistical analysis was performed using GraphPad Prism 8.0. Data on the graphs were presented as mean ± SD. One-way Analysis of Variance (ANOVA) was used to analyze the difference between different treatments and differences were considered significant at a *p*-value of < 0.05.

## Supporting information

S1 Fig PTC readthrough in a cell-free translation assay. WT (A) and R213X *TP53* (B) mRNAs were translated in vitro in the presence of G418 at the indicated concentrations and equal amounts of protein were subjected to automated capillary electrophoresis western analysis. The data show the average percentage of full-length p53 relative to the maximum value observed in either WT or R213X for 3 biological replicates. Asterisks show statistically significant differences between treated and untreated cells (*p*-value < 0.05; mean ± SEM). The numerical data underlying the plots can be found in S6 Data.
(TIF)

S2 Fig Y-320 enhances PTC readthrough by gentamicin in HDQ-P1 cells. HDQ-P1 cells were exposed to the indicated compounds for 72 h and subjected to automated capillary electrophoresis western analysis. The displayed images are “pseudo blots” of detected chemiluminescence of bound p53 and vinculin antibodies. The area under the full-length p53 peaks was normalized to the vinculin loading control to provide lane-to-lane comparison. These numbers are displayed under the lanes. Uncropped images of automated capillary electrophoresis western analysis are provided in S1 Raw Images. FL-p53, full-length p53; TR-p53, truncated p53.
(TIF)

S3 Fig Y-320 does not increase COL17A1 levels in HaCat cells. HaCat cells were exposed to the indicated compounds for 72 h. Cells were lysed and equal amounts of protein were subjected to automated capillary electrophoresis western analysis. Shown are pseudo blots of chemiluminescence of bound COL17A1 and beta-actin antibodies. The area under the full-length COL17A1 peaks was first normalized to the beta-actin loading control and then divided to that of 30 μM G418 to provide lane-to-lane comparison. These numbers are displayed under the lanes. Uncropped images of automated capillary electrophoresis western analysis are provided in S1 Raw Images.
(TIF)

S4 Fig Effect of Y-320 on *TP53* mRNA and full-length p53 in HDQ-P1 cells. HDQ-P1 cells were exposed to the indicated concentrations of compounds for 48 h and analyzed for levels of *TP53* mRNA (A) and full-length p53 (B). (A) *TP53* mRNA was measured using qPCR and expressed relative to G418. Asterisks show statistically significant differences (*p*-value < 0.05; mean ± SD; *n =* 3). (B) Full-length p53 was measured using automated capillary electrophoresis western analysis, normalized to vinculin as a loading control, and expressed relative to p53 levels in cells exposed to G418. The numerical data underlying the plots can be found in S6 Data.
(TIF)

S5 Fig PTC readthrough in a cell-free translation assay. R213X *TP53* mRNA was translated in vitro in the presence of G418, Y-320, or G418 + Y-320. Equal amounts of protein were subjected to automated capillary electrophoresis western analysis. The data are displayed as “pseudo blots” of bound p53 antibody chemiluminescence. Full-length p53 (FL-p53) chemiluminescence is expressed relative to the maximum value and shown under each lane. Uncropped images of automated capillary electrophoresis western analysis are provided in S1 Raw Images.
(TIF)

S6 Fig Y-320 increases protein synthesis in WT fibroblasts and H1299-R213X cells. (A) Detection of protein synthesis in WT fibroblasts. Fibroblasts treated with Y-320 for 24 h were exposed to HPG reagent, fluorescently labeled using Alexa Fluor 488 azide and imaged using automated immunofluorescence microscopy. (B) Detection of protein synthesis in H1299-R213X. Cells treated for 24 h were exposed to puromycin and labeled with anti-puromycin AF488 antibody. Data were collected by automated fluorescence microscopy. Asterisks show statistically significant differences between treated and untreated cells (*p*-value < 0.05; mean ± SD; *n =* 4). The numerical data underlying the plots can be found in S6 Data.
(TIF)

S7 Fig Effect of Y-320 on cell number in HDQ-P1 cells. HDQ-P1 cells were exposed to Y-320 for 48 h, fixed and labeled for nuclei using Hoechst nuclear stain for 20 min. Data were collected by automated fluorescence microscopy (mean ± SD; *n* = 4). The numerical data underlying the plot can be found in S6 Data.
(TIF)

S8 Fig Effects of Y-320 on cell viability in HDQ-P1 cells. HDQ-P1 cells were incubated with different concentrations of Y-320 alone or combined with 200 μM G418 for 24 h (A) or 48 h (B). Cell viability was measured using the MTT assay (mean ± SD; *n* = 4). The numerical data underlying the plots can be found in S6 Data.
(TIF)

S9 Fig Effect of Y-320 on translation elongation rate. HDQ-P1 cells were exposed or not to 2 μM Y-320 for 48 h. At the end of treatment, cells were exposed to harringtonine (5 μg/ml) for different times (minutes) prior to incubation with puromycin (10 μg/ml) for 10 min. Cells were fixed and puromycin signal was detected with anti-puromycin AF488 antibody. Data were collected from 2 biological and 6 total technical replicates by automated fluorescence microscopy. Datasets were subjected to nonlinear regression using GraphPad Prism software (the 95% confidence interval is shown in blue and gray for Y-320 treated and control cells, respectively). The numerical data underlying the plot can be found in S6 Data.
(TIF)

S10 Fig Y-320 does not increase translation initiation. HDQ-P1 (A) and H1299-R213X (B) cells were exposed to the indicated compounds for 48 h and 24 h, respectively. Shown are pseudo blots of chemiluminescence of bound eIF4G, phospho-eIF4B, and vinculin antibodies. Uncropped images of automated capillary electrophoresis western analyses are provided in S1 Raw Images.
(TIF)

S11 Fig Up-regulation of CXC chemokines by Y-320 verified by qPCR. HDQ-P1 cells exposed to 1 μM Y-320 for 48 h were analyzed for formation of *CXCL* mRNAs relative to untreated cells. Asterisks show statistically significant differences between treated and untreated cells (*p*-value < 0.05; mean ± SD; *n =* 6). The numerical data underlying the plot can be found in S6 Data.
(TIF)

S12 Fig Stimulation of ribosome biogenesis is not observed with other readthrough enhancers. HDQ-P1 cells were treated with the indicated concentrations of readthrough enhancers without or with G418 for 48 h and fluorescently labeled with anti-p53 (A), anti-rRNA (B), or anti-rpS6 (C) antibodies. Data were collected by automated quantitative fluorescence microscopy (mean ± SD; A, *n* = 3; B and C, *n* = 4). The numerical data underlying the plots can be found in S6 Data.
(EPS)

S1 Table Screening results. HDQ-P1 cells were exposed to the compounds in combination with 50 μM G418. After 72 h, cells were fluorescently labeled using anti-p53 antibody, and *TP53* PTC readthrough was assessed using quantitative automated p53 immunofluorescence microscopy. The number of cells measured in 12 fields in each well (Valid Object Count) and p53 immunofluorescence (MEAN-CircAvgInten) are displayed. The numbers for Valid Object Count and p53 MEAN-CircAvgInten of untreated cells (mean ± SD; *n* > 100) were 2,272 ± 714 and 29.66 ± 4.36, respectively. The numbers for Valid Object Count and p53 Mean-CircAvgInten of G418-treated cells (mean ± SD; *n* > 100) were 2,284 ± 708 and 31.32 ± 4.12, respectively. Compounds with high toxicity (<2,000 cells) were removed from the list and the remaining compounds were sorted by p53 signal intensity.
(XLSX)

S2 Table List of primers used for q-PCR. (DOCX)

S1 Data Numerical raw data. All numerical raw data associated with Fig 3A–3C. File contains multiple tabs with labels corresponding to the relevant figure.
(XLSX)

S2 Data Numerical raw data. All numerical raw data associated with Fig 4A–4C. File contains multiple tabs with labels corresponding to the relevant figure.
(XLSX)

S3 Data Numerical raw data. All numerical raw data associated with Fig 5A, 5B and 5E–5G. File contains multiple tabs with labels corresponding to the relevant figure.
(XLSX)

S4 Data Numerical raw data. All numerical raw data associated with Fig 6.
(XLSX)

S5 Data Numerical raw data. All numerical raw data associated with Fig 7B and 7C. File contains multiple tabs with labels corresponding to the relevant figure.
(XLSX)

S6 Data Numerical raw data. All numerical raw data associated with S1A, S1B, S4A, S4B, S6A, S6B, S7, S8A, S8B, S9, S11 and S12A–S12C Figs. File contains multiple tabs with labels corresponding to the relevant figure.
(XLSX)

S1 Raw Images Uncropped wetern blots from all main and supporting information figures. (PDF)

## Abbreviations

4EBP1: 4E-binding protein 1
BSA: bovine serum albumin
DMD: Duchenne Muscular Dystrophy
FBS: fetal bovine serum
HPG: L-homopropargylglycine
JEB: Junctional Epidermolysis Bullosa
K-SFM: keratinocyte serum-free medium
mAb: monoclonal antibody
MEF: mouse embryonic fibroblast
mTOR: mammalian target of rapamycin
NMD: nonsense mediated decay
PBS: phosphate-buffered saline
PTC: premature termination codon
RDEB: recessive dystrophic EB
RNA-seq: RNA sequencing
rRNA: ribosomal RNA
S6K: S6 kinase
WT: wild-type
