# Supplementary figures and images for: Small molecule Y-320 stimulates ribosome biogenesis, protein synthesis, and aminoglycoside-induced premature termination codon readthrough
Source: PLoS Biol. 2021 May 3;19(5):e3001221. doi: 10.1371/journal.pbio.3001221 (PMC8118496; doi:10.1371/journal.pbio.3001221)

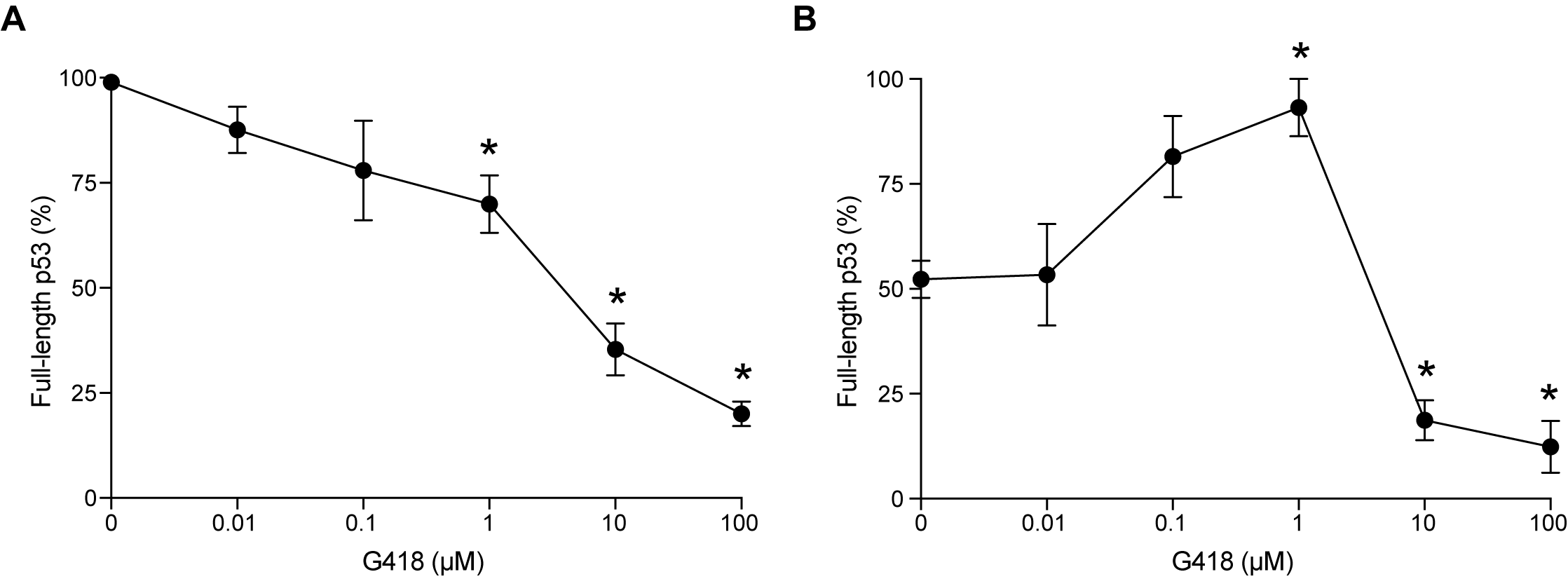

Supplement: S1 Fig — WT (A) and R213X TP53 (B) mRNAs were translated in vitro in the presence of G418 at the indicated concentrations and equal amounts of protein were subjected to automated capillary electrophoresis western analysis. The data show the average percentage of full-length p53 relative to the maximum value observed in either WT or R213X for 3 biological replicates. Asterisks show statistically significant differences between treated and untreated cells (p-value < 0.05; mean ± SEM). The numerical data underlying the plots can be found in S6 Data. (TIF) [file pbio.3001221.s001.tif]

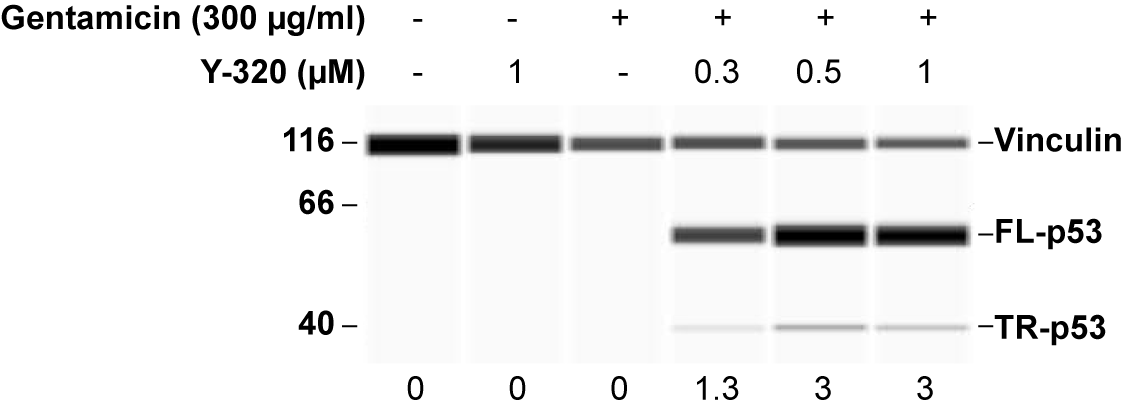

Supplement: S2 Fig — HDQ-P1 cells were exposed to the indicated compounds for 72 h and subjected to automated capillary electrophoresis western analysis. The displayed images are “pseudo blots” of detected chemiluminescence of bound p53 and vinculin antibodies. The area under the full-length p53 peaks was normalized to the vinculin loading control to provide lane-to-lane comparison. These numbers are displayed under the lanes. Uncropped images of automated capillary electrophoresis western analysis are provided in S1 Raw Images. FL-p53, full-length p53; TR-p53, truncated p53. (TIF) [file pbio.3001221.s002.tif]

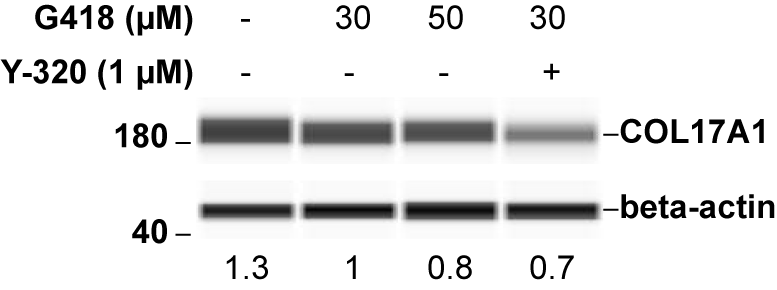

Supplement: S3 Fig — HaCat cells were exposed to the indicated compounds for 72 h. Cells were lysed and equal amounts of protein were subjected to automated capillary electrophoresis western analysis. Shown are pseudo blots of chemiluminescence of bound COL17A1 and beta-actin antibodies. The area under the full-length COL17A1 peaks was first normalized to the beta-actin loading control and then divided to that of 30 μM G418 to provide lane-to-lane comparison. These numbers are displayed under the lanes. Uncropped images of automated capillary electrophoresis western analysis are provided in S1 Raw Images. (TIF) [file pbio.3001221.s003.tif]

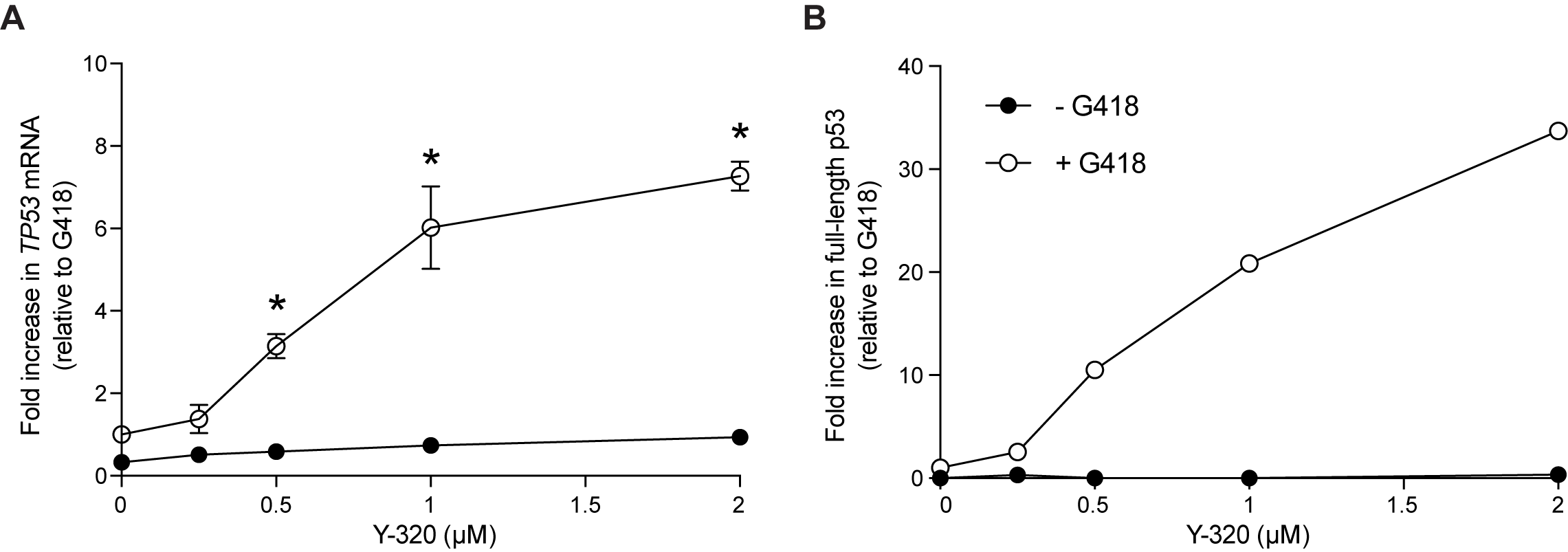

Supplement: S4 Fig — HDQ-P1 cells were exposed to the indicated concentrations of compounds for 48 h and analyzed for levels of TP53 mRNA (A) and full-length p53 (B). (A) TP53 mRNA was measured using qPCR and expressed relative to G418. Asterisks show statistically significant differences (p-value < 0.05; mean ± SD; n = 3). (B) Full-length p53 was measured using automated capillary electrophoresis western analysis, normalized to vinculin as a loading control, and expressed relative to p53 levels in cells exposed to G418. The numerical data underlying the plots can be found in S6 Data. (TIF) [file pbio.3001221.s004.tif]

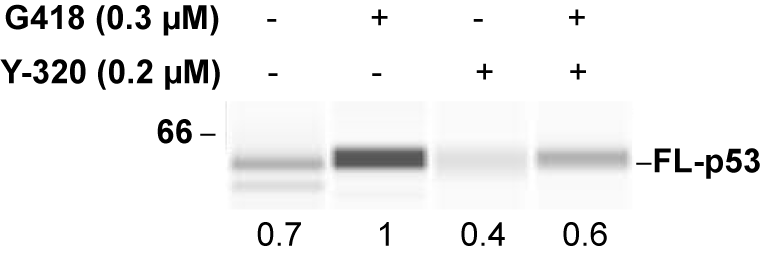

Supplement: S5 Fig — R213X TP53 mRNA was translated in vitro in the presence of G418, Y-320, or G418 + Y-320. Equal amounts of protein were subjected to automated capillary electrophoresis western analysis. The data are displayed as “pseudo blots” of bound p53 antibody chemiluminescence. Full-length p53 (FL-p53) chemiluminescence is expressed relative to the maximum value and shown under each lane. Uncropped images of automated capillary electrophoresis western analysis are provided in S1 Raw Images. (TIF) [file pbio.3001221.s005.tif]

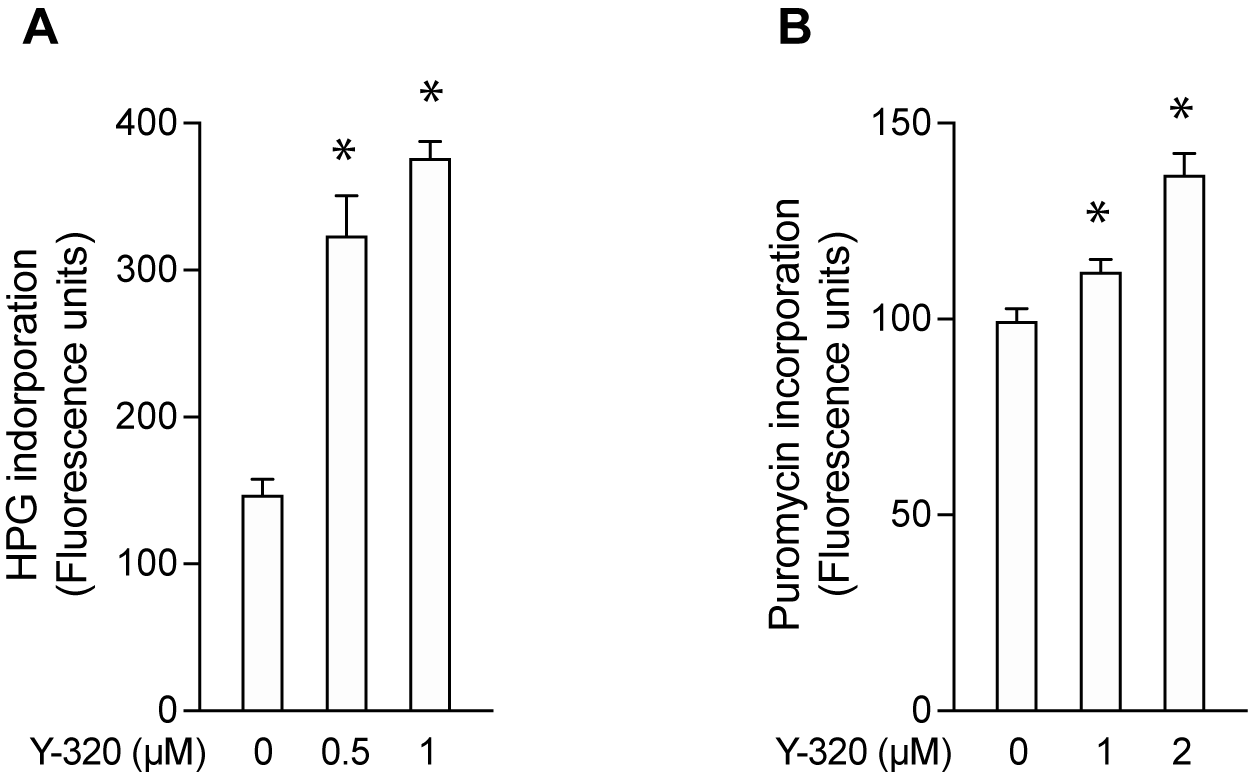

Supplement: S6 Fig — (A) Detection of protein synthesis in WT fibroblasts. Fibroblasts treated with Y-320 for 24 h were exposed to HPG reagent, fluorescently labeled using Alexa Fluor 488 azide and imaged using automated immunofluorescence microscopy. (B) Detection of protein synthesis in H1299-R213X. Cells treated for 24 h were exposed to puromycin and labeled with anti-puromycin AF488 antibody. Data were collected by automated fluorescence microscopy. Asterisks show statistically significant differences between treated and untreated cells (p-value < 0.05; mean ± SD; n = 4). The numerical data underlying the plots can be found in S6 Data. (TIF) [file pbio.3001221.s006.tif]

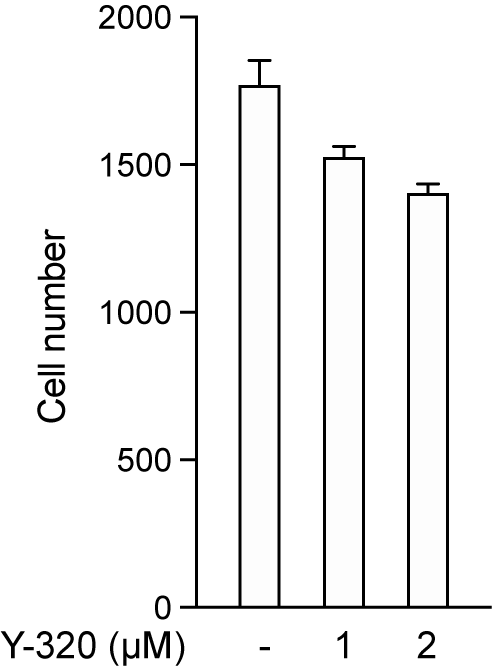

Supplement: S7 Fig — HDQ-P1 cells were exposed to Y-320 for 48 h, fixed and labeled for nuclei using Hoechst nuclear stain for 20 min. Data were collected by automated fluorescence microscopy (mean ± SD; n = 4). The numerical data underlying the plot can be found in S6 Data. (TIF) [file pbio.3001221.s007.tif]

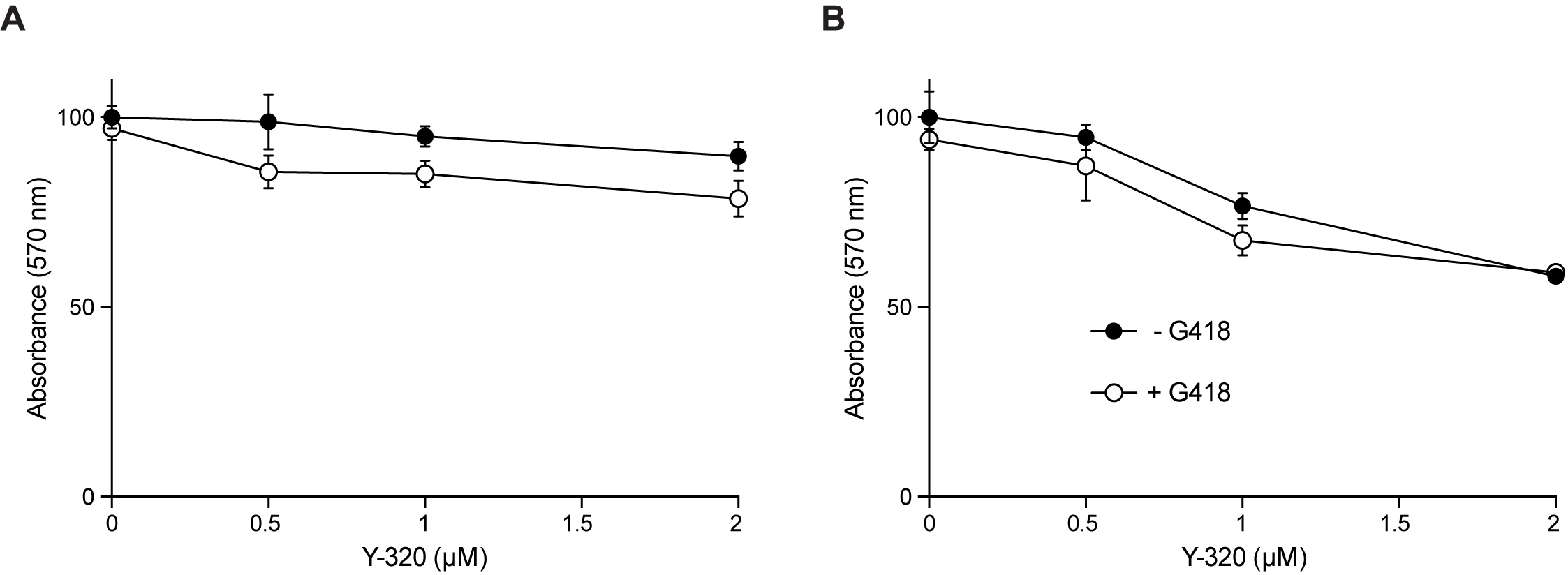

Supplement: S8 Fig — HDQ-P1 cells were incubated with different concentrations of Y-320 alone or combined with 200 μM G418 for 24 h (A) or 48 h (B). Cell viability was measured using the MTT assay (mean ± SD; n = 4). The numerical data underlying the plots can be found in S6 Data. (TIF) [file pbio.3001221.s008.tif]

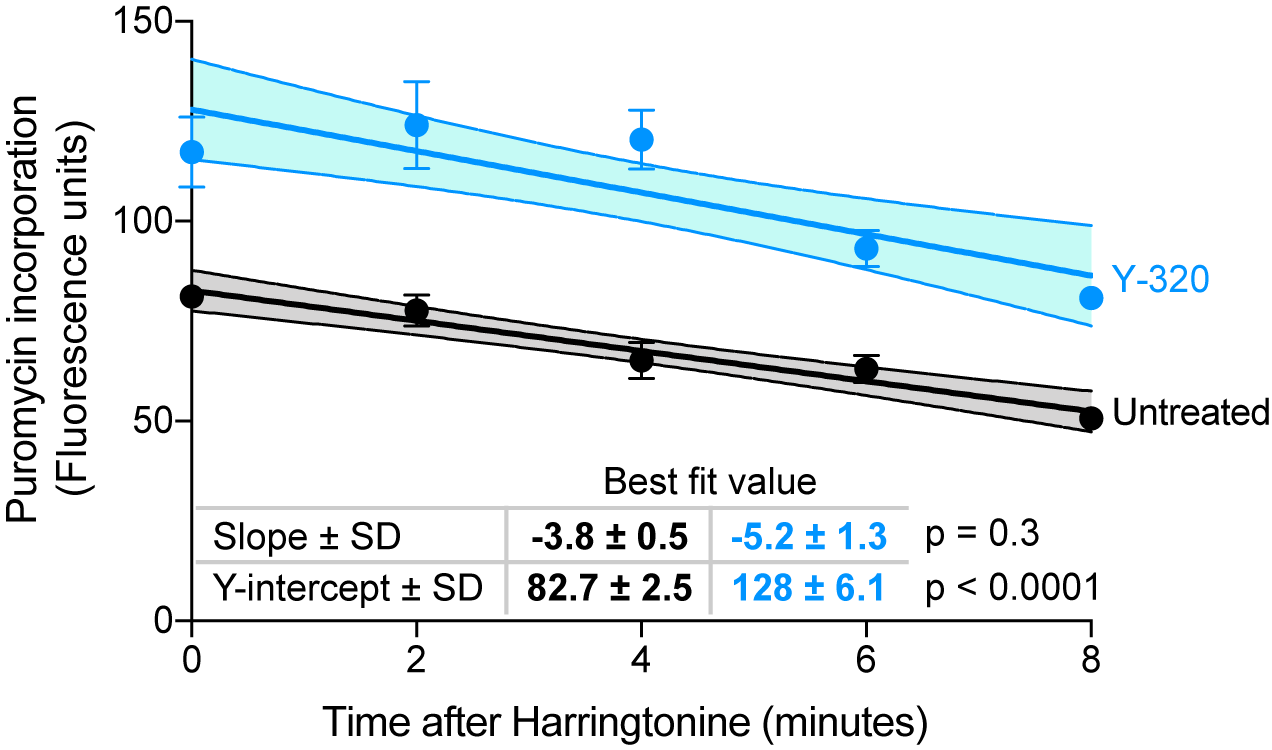

Supplement: S9 Fig — HDQ-P1 cells were exposed or not to 2 μM Y-320 for 48 h. At the end of treatment, cells were exposed to harringtonine (5 μg/ml) for different times (minutes) prior to incubation with puromycin (10 μg/ml) for 10 min. Cells were fixed and puromycin signal was detected with anti-puromycin AF488 antibody. Data were collected from 2 biological and 6 total technical replicates by automated fluorescence microscopy. Datasets were subjected to nonlinear regression using GraphPad Prism software (the 95% confidence interval is shown in blue and gray for Y-320 treated and control cells, respectively). The numerical data underlying the plot can be found in S6 Data. (TIF) [file pbio.3001221.s009.tif]

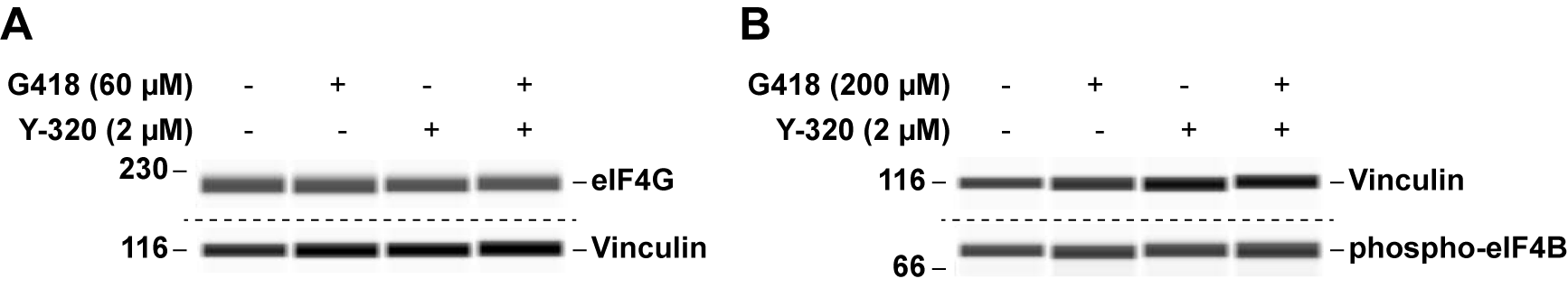

Supplement: S10 Fig — HDQ-P1 (A) and H1299-R213X (B) cells were exposed to the indicated compounds for 48 h and 24 h, respectively. Shown are pseudo blots of chemiluminescence of bound eIF4G, phospho-eIF4B, and vinculin antibodies. Uncropped images of automated capillary electrophoresis western analyses are provided in S1 Raw Images. (TIF) [file pbio.3001221.s010.tif]

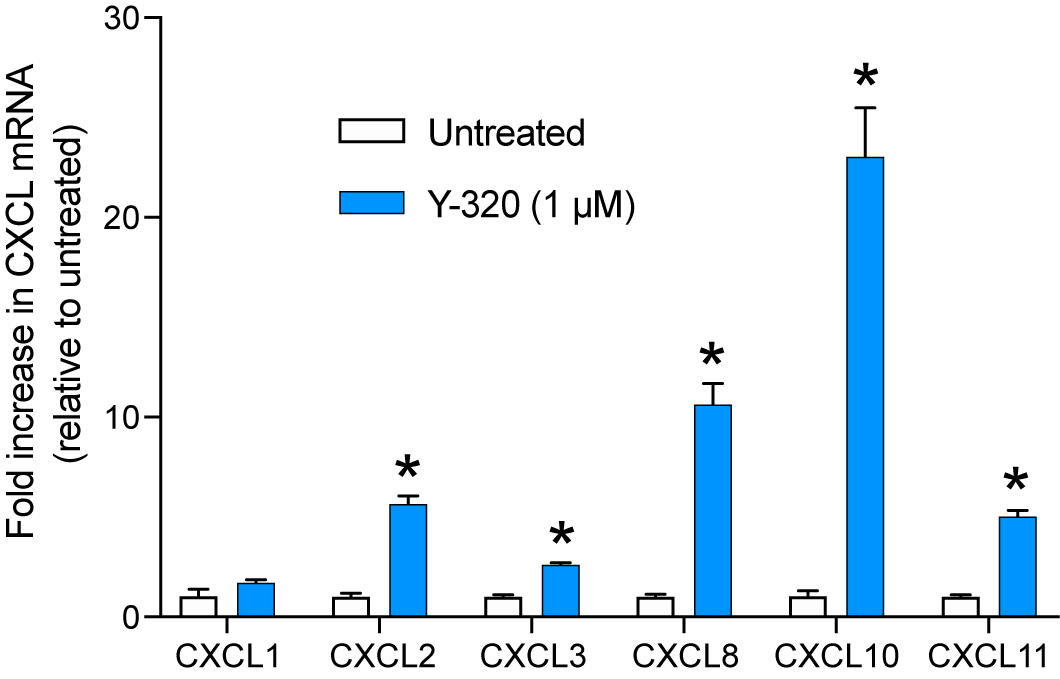

Supplement: S11 Fig — HDQ-P1 cells exposed to 1 μM Y-320 for 48 h were analyzed for formation of CXCL mRNAs relative to untreated cells. Asterisks show statistically significant differences between treated and untreated cells (p-value < 0.05; mean ± SD; n = 6). The numerical data underlying the plot can be found in S6 Data. (TIF) [file pbio.3001221.s011.tif]
