# Supplementary material for: Small molecule Y-320 stimulates ribosome biogenesis, protein synthesis, and aminoglycoside-induced premature termination codon readthrough
Source: PLoS Biol. 2021 May 3;19(5):e3001221. doi: 10.1371/journal.pbio.3001221 (PMC8118496; doi:10.1371/journal.pbio.3001221)
Supplement: S2 Table — (DOCX) [file pbio.3001221.s014.docx]

**S2 Table**

| Gene name | Primer sequence (5’ → 3’) |
| --- | --- |
|  |  |
| HPRT1 | Forward CCCTGGCGTCGTGATTAGTG |
|  | Reverse CACCCTTTCCAAATCCTCAGCA |
|  |  |
| TP53 | Forward CGCTTCGAGATGTTCCGAGA |
|  | Reverse CTTCAGGTGGCTGGAGTGAG |
|  |  |
| CXCL1 | Forward CTGGCTTAGAACAAAGGGGCT |
|  | Reverse TAAAGGTAGCCCTTGTTTCCCC |
|  |  |
| CXCL2 | Forward GAAAGCTTGTCTCAACCCCG |
|  | Reverse TGGTCAGTTGGATTTGCCATTTT |
|  |  |
| CXCL3 | Forward AAAAGATACTGAACAAGGGGAGCA |
|  | Reverse CTCTGGTAAGGGCAGGGACC |
|  |  |
| CXCL8 | Forward GAAGTTTTTGAAGAGGGCTGAGA |
|  | Reverse TGCTTGAAGTTTCACTGGCATC |
|  |  |
| CXCL10 | Forward AAGTGGCATTCAAGGAGTACCT |
|  | Reverse ACACGTGGACAAAATTGGCT |
|  |  |
| CXCL11 | Forward GCTACAGTTGTTCAAGGCTTCC |
|  | Reverse GCTTTTACCCCAGGGCCTAT |
