# Supplementary material for: Small molecule Y-320 stimulates ribosome biogenesis, protein synthesis, and aminoglycoside-induced premature termination codon readthrough
Source: PLoS Biol. 2021 May 3;19(5):e3001221. doi: 10.1371/journal.pbio.3001221 (PMC8118496; doi:10.1371/journal.pbio.3001221)
Supplement: S1 Raw Images — (PDF) [file pbio.3001221.s021.pdf]

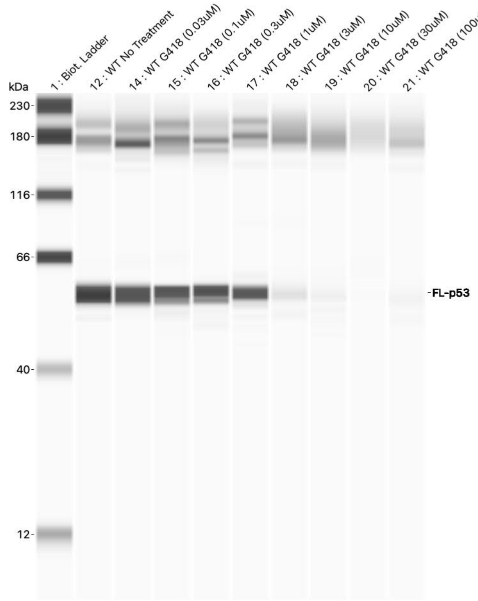

Fig. 1-WT

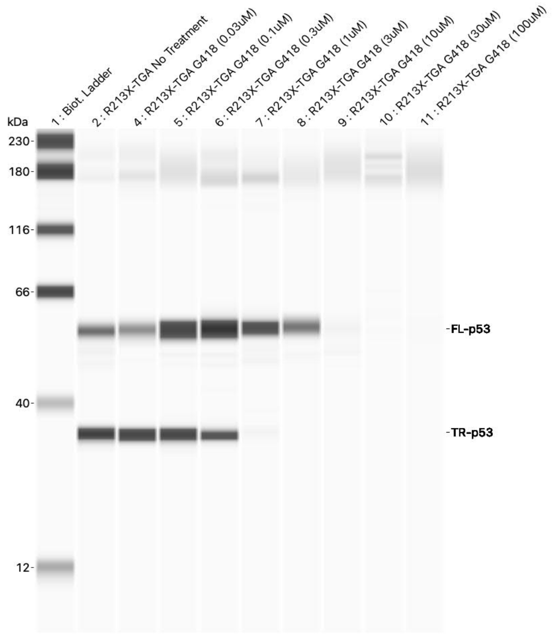

Fig. 1-R213X

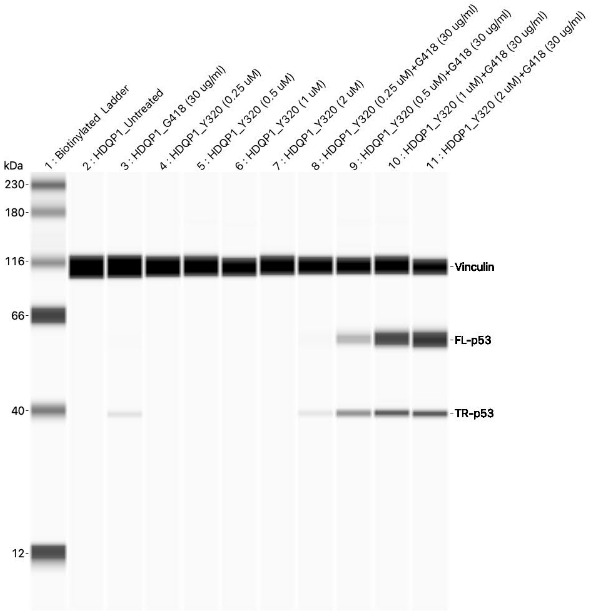

Fig. 2A

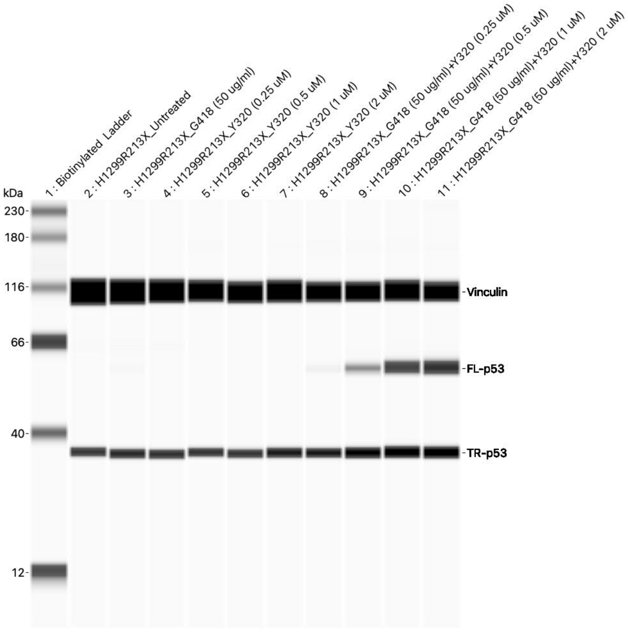

Fig. 2B

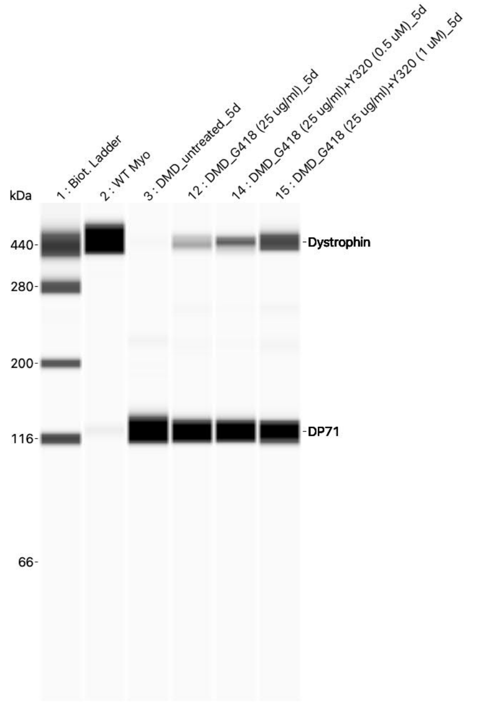

Fig. 2C
DP71 is a shorter
dystrophin isoform.

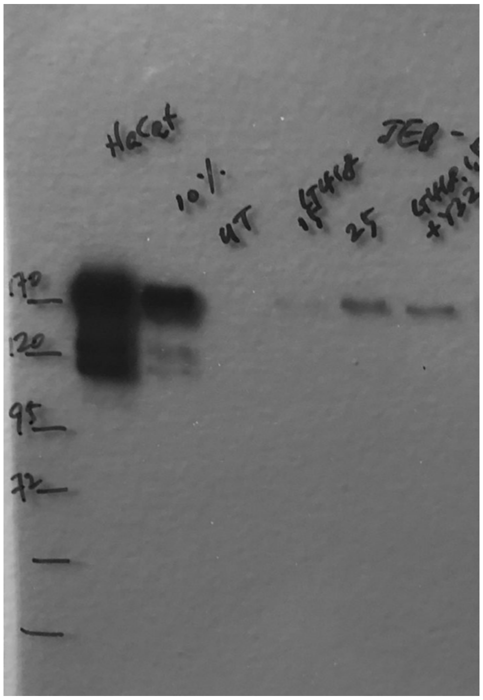

Fig.2D

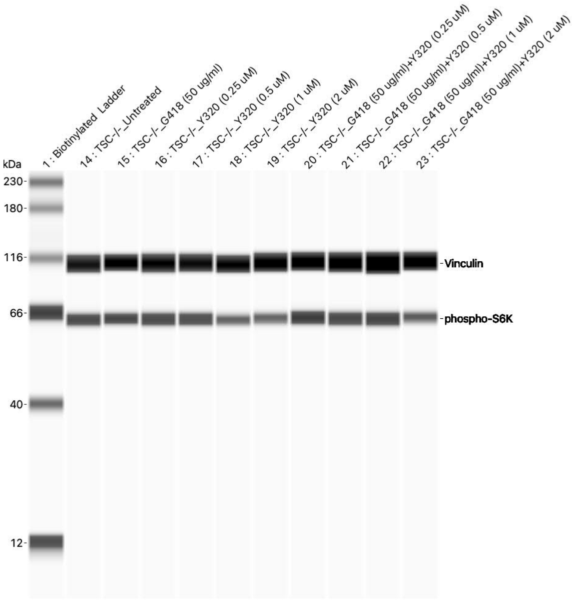

Fig. 5C
Y-320 at 0.25 μM is not included
in the manuscript.

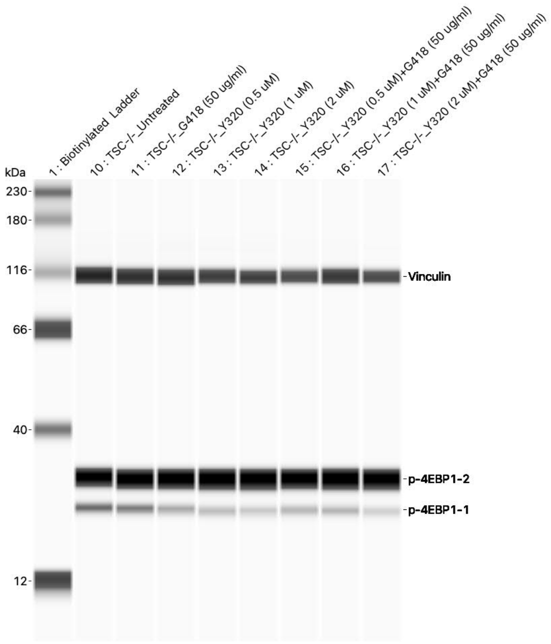

Fig. 5D

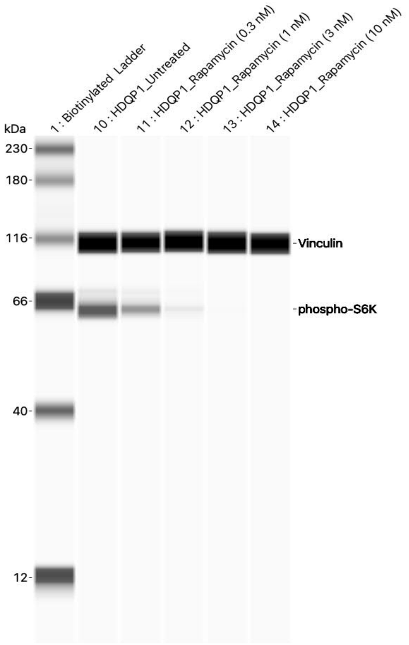

Fig. 5H

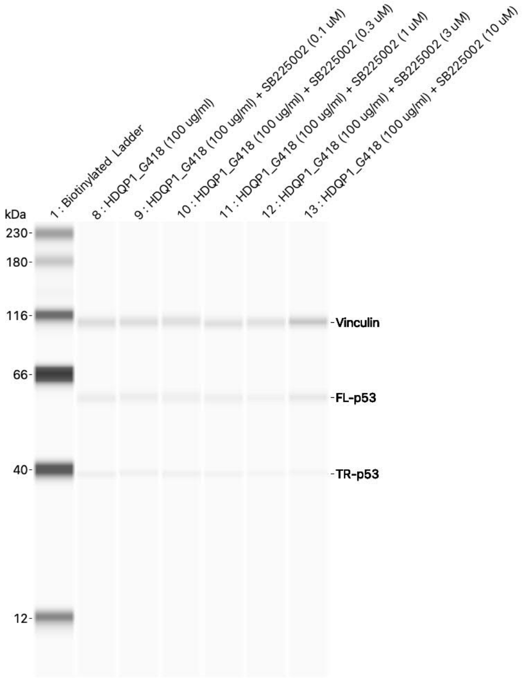

Fig. 7D top panel

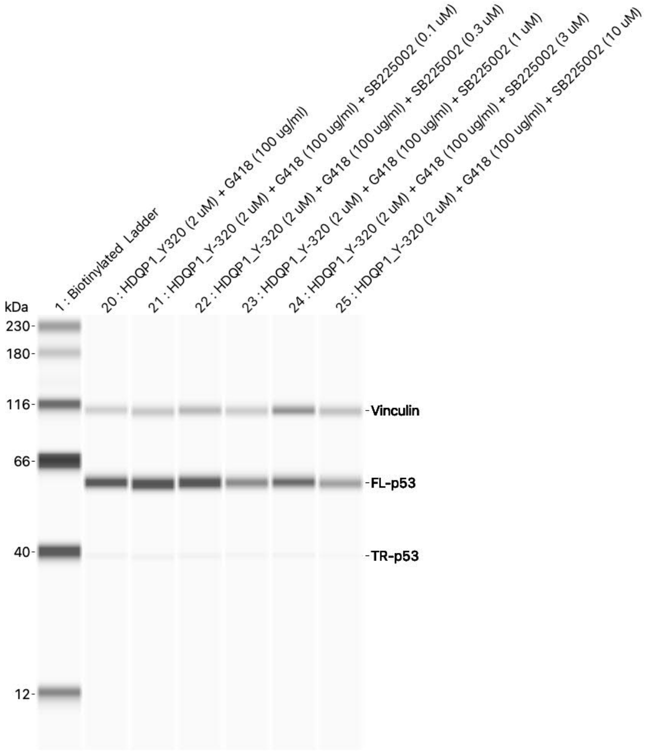

Fig. 7D bottom panel

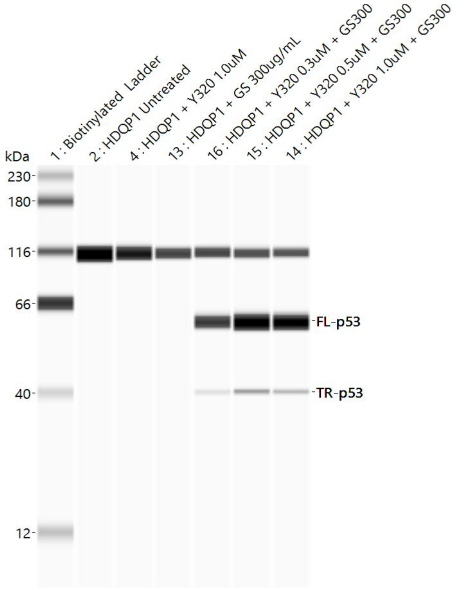

S2 Fig.
GS: Gentamicin

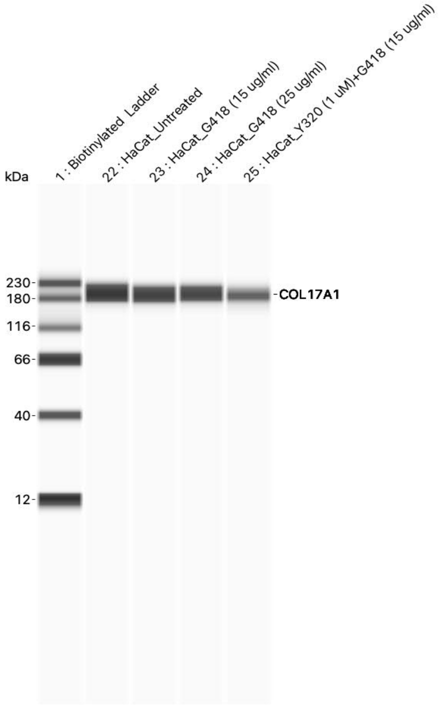

S3 Fig. COL17A1

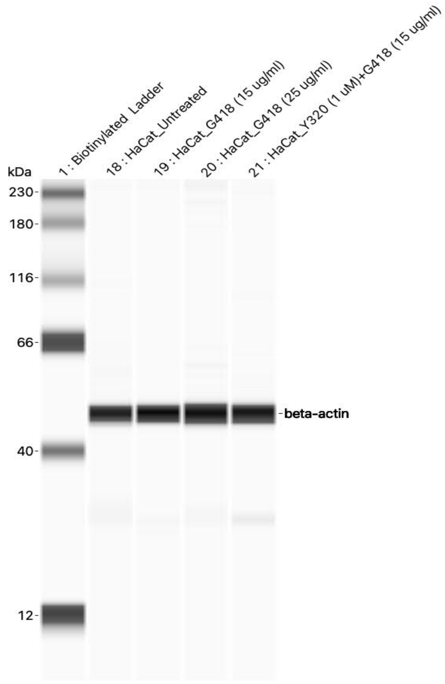

S3 Fig. b-actin

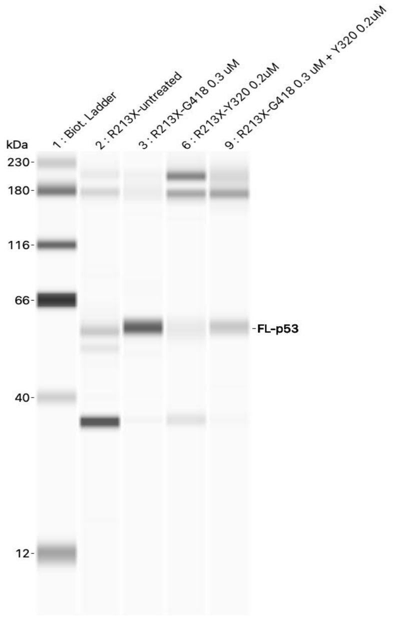

S5 Fig.

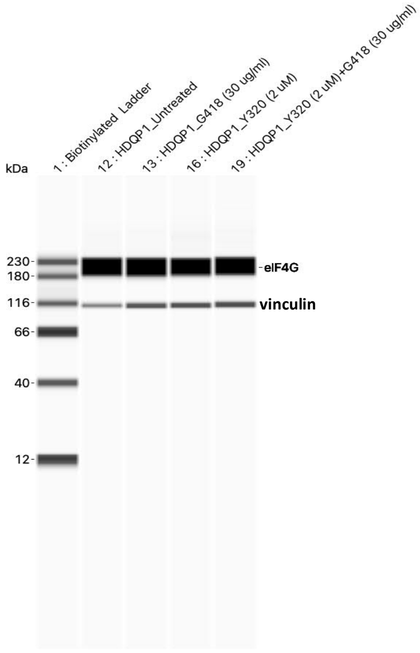

S10A Fig.

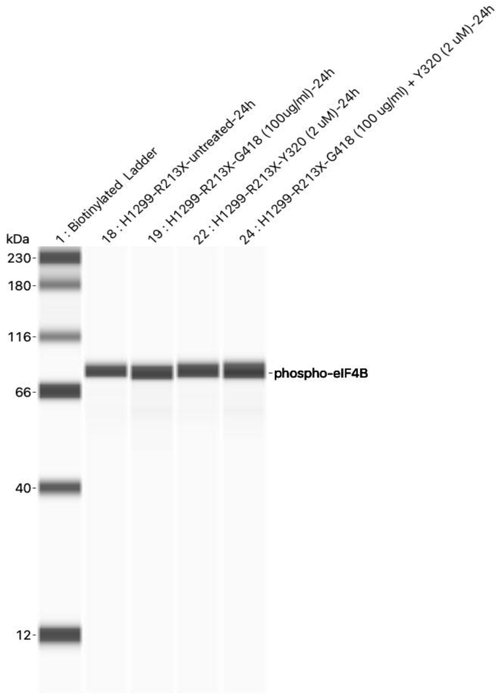

S10B Fig. p-eIF4B

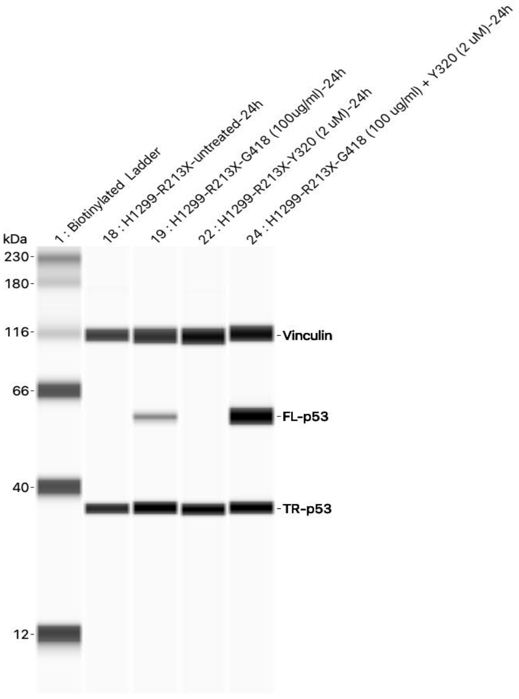

S10B Fig. Vinculin
These are the same samples as the left panel. From this image, only vinculin is included in the manuscript.
